# Presence of *Trifolium repens* Promotes Complementarity of Water Use and N Facilitation in Diverse Grass Mixtures

**DOI:** 10.3389/fpls.2016.00538

**Published:** 2016-04-26

**Authors:** Pauline Hernandez, Catherine Picon-Cochard

**Affiliations:** INRA, UREP, Grassland Ecosystem ResearchClermont-Ferrand, France

**Keywords:** deep root, functional diversity, leaf area, legume, minirhizotron, over-yielding, water use efficiency

## Abstract

Legume species promote productivity and increase the digestibility of herbage in grasslands. Considerable experimental data also indicate that communities with legumes produce more above-ground biomass than is expected from monocultures. While it has been attributed to N facilitation, evidence to identify the mechanisms involved is still lacking and the role of complementarity in soil water acquisition by vertical root differentiation remains unclear. We used a 20-months mesocosm experiment to investigate the effects of species richness (single species, two- and five-species mixtures) and functional diversity (presence of the legume *Trifolium repens*) on a set of traits related to light, N and water use and measured at community level. We found a positive effect of *Trifolium* presence and abundance on biomass production and complementarity effects in the two-species mixtures from the second year. In addition the community traits related to water and N acquisition and use (leaf area, N, water-use efficiency, and deep root growth) were higher in the presence of *Trifolium*. With a multiple regression approach, we showed that the traits related to water acquisition and use were with N the main determinants of biomass production and complementarity effects in diverse mixtures. At shallow soil layers, lower root mass of *Trifolium* and higher soil moisture should increase soil water availability for the associated grass species. Conversely at deep soil layer, higher root growth and lower soil moisture mirror soil resource use increase of mixtures. Altogether, these results highlight N facilitation but almost soil vertical differentiation and thus complementarity for water acquisition and use in mixtures with *Trifolium*. Contrary to grass-*Trifolium* mixtures, no significant over-yielding was measured for grass mixtures even those having complementary traits (short and shallow vs. tall and deep). Thus, vertical complementarity for soil resources uptake in mixtures was not only dependant on the inherent root system architecture but also on root plasticity. We also observed a time-dependence for positive complementarity effects due to the slow development of *Trifolium* in mixtures, possibly induced by competition with grasses. Overall, our data underlined that soil water resource was an important driver of over-yielding and complementarity effects in *Trifolium*-grass mixtures.

## Introduction

Legume species promote productivity and increase the digestibility and protein content of herbage in grasslands managed with low fertilizer inputs (Frame, 1986; Jarvis et al., 1996). Considerable experimental evidence also indicates that communities with legumes produce more above-ground biomass than is expected from monocultures (Loreau et al., 2001; Schmid, 2002; Temperton et al., 2007; Dybzinski et al., 2008; Marquard et al., 2009; Roscher et al., 2012). Positive effects of legumes are generally attributed to increases in soil nitrogen availability through atmospheric N_2_ fixation (Hille Ris Lambers et al., 2004; Cardinale et al., 2007). However, little is known about the role of soil water resource on over-yielding effect in mixtures with legume species, despite soil water availability could also play an important role for the initiation of diversity effect (Silvertown et al., 1999; Nippert and Knapp, 2007).

Together with N facilitation by legume, niche complementarity has been proposed as main underlying mechanisms for over-yielding in diverse mixtures. Such complementarity may occur through differences in resource uptake in time, chemical form and especially in space between species (Kahmen et al., 2006; von Felten et al., 2009), allowing a more exhaustive exploitation of the soil resource (Dimitrakopoulos and Schmid, 2004; Fargione and Tilman, 2005; von Felten and Schmid, 2008). Inter-specific complementarity of plant traits linked to resource acquisition should therefore enhance productivity in mixtures compared with assemblages of species with similar traits. Theoretically, it has been argued that assembling different rooting depth species in mixtures should lead to vertical niche differentiation (Berendse, 1979). In grassland or savannas, communities with deep tap rooted dicots and shallow fibrous rooted grasses are a good example of such below-ground vertical niche differentiation (Nippert and Holdo, 2015). However, the existence of vertical complementarity for below-ground resources uptake in mixtures is a controversial assumption because root plasticity more than inherent different rooting distribution should be taken into account (Mommer et al., 2010; Mueller et al., 2013). Vertical complementarity for soil water uptake has received little attention in grass-legume mixtures (Grieu et al., 2001; Verheyen et al., 2008) and should be further explored as determinant of over-yielding.

The niche complementarity mechanism appears to strongly depend on the functional traits of the species, especially related to below-ground resource acquisition and use. Thus, it has been proposed that extensive analyses of diversity-productivity relationship should focus on the functional traits composition and diversity of communities (Mason et al., 2005). Community-weighted means of trait values, quantifying the dominant trait values in a community, and functional trait diversity, quantifying the distribution of trait values among species, have been shown to jointly explain variations in above-ground productivity in semi-natural grasslands (Díaz et al., 2007; Mokany et al., 2008; Schumacher and Roscher, 2009). In case of over-yielding in grass-legume mixtures, using this approach on traits composition and diversity indices related to specific functions and resources can lead to an efficient identification and quantification of facilitation and complementarity mechanisms involved.

Despite many evidences on the importance of legume for over-yielding establishment, the effects of legume abundance fluctuations have been scarcely taken into account. Indeed, the proportion of legume in sown mixtures and in permanent grasslands fluctuates, both from year to year and within single growth periods (Frame, 1986), which have been attributed to effects of abiotic and biotic factors on N_2_ fixation (Soussana and Tallec, 2010). White clover (*Trifolium repens* called thereafter *Trifolium*) is one of the most effective N_2_ fixing species in mesic pastures (Haynes, 1980). In fertile grasslands its development could be slowed, because large biomass accumulation of grasses leads to conspicuous and asymmetric competition for light (Schwinning and Weiner, 1998). Moreover, at below-ground level, it is assumed that species capture the resource in proportion to their root length density (Craine and Dybzinski, 2013), thus competition for water and nutrients induced by grasses having longer, thinner, and more finely branched roots than legumes (Evans, 1977) is to consider in diverse mixtures with legume species. However, in case of *Trifolium* this assumption is challenged as this species is able to take up more water than rye grass and from deeper soil layer with less dense shallow roots but with deep tap root system (Høgh-Jensen and Schjoerring, 1997; Grieu et al., 2001).

In the present work, we studied under well-watered conditions for 20 months, a set of traits related to light, N and water use as predictors of biomass production and diversity effects of mixtures with or without the presence of a legume species *T. repens* (**Table 1**). By using models selection, we set out to identify and rank which leaf and root traits better explain biomass, over-yielding and complementarity effect in mixture and therefore which resources are mainly involved in these responses. We assume that functional diversity through *Trifolium* presence is more important than species richness to explain biomass production and diversity effects. Thus our main hypothesis is that over-yielding only occurs in grass-*Trifolium* mixture leading to the highest biomass production. We suppose that the underlying mechanisms are: (1) N facilitation leading to higher N yield and leaf N concentration of community and associated grass, respectively; (2) complementarity through vertical differentiation for soil water uptake. We expect higher deep root growth because of root plasticity, whatever the grass composition in the mixture, leading to a more efficient use of resources along the soil profile and higher transpiration. Finally, time-dependence of these diversity effects, driven by possible change of *Trifolium* abundance, is also explored over several periods.

**Table 1:** List of traits, their potential associated function, link with resource, period of measurement, unit and calculation of community-weighted mean (CWM) and functional diversity (FD_Q_).

Functional traits	Functions	Resources	Measurement time	Unit	CWM	FD_Q_
Maximum height growth (H.growth)	Light acquisition	C/light	Seventeen growth periods between spring in year 1 (DOY 136) and spring in year 2 (DOY 156)	cm day^-1^	Yes	Yes
Above-ground biomass in the “light” layer (Biom st1)	Light acquisition	C/light	Two periods of aboveground production: spring year 1 and spring year 2	%	Yes	Yes
Leaf area (L.area)	Light acquisition; indicator of evapotranspiration	Light/water	Six periods of above-ground production	m^2^ pot^-1^	Yes	Yes
Leaf carbon isotopic composition (δ^13^C)	Proxy of water use efficiency	Light/C/water	Two periods of above-ground production: spring year 1 and spring year 2	aaa	Yes	Yes
Leaf dry matter content (LDMC)	Density of leaf tissue; conservation of resource	C/N	Two dates: spring in year 1 (DOY 137) and spring in year 2 (DOY 157)	mg g^-1^	Yes	Yes
Nitrogen content of community (N)	Photosynthetic rate; N use	N	Six dates of forage harvest	%	Yes	Yes
Nitrogen yield/ET (Nyield/ET)	Balance of N use to water use	N/water	Six periods of above-ground production	gN kgH_2_O^-1^	Yes	No
Maximum deep root growth (R.growth)	Capacity to water and nutrient use at deep soil layer	N/water	Twenty two growth periods between DOY 127 year 1 and DOY 147 year 2	mm cm^-2^ day^-1^	Yes	No
Integrated water use efficiency (WUE)	Biomass production and water use	C/water	Six periods of above-ground production	gDM kg^-1^H_2_O	Yes	No
Soil relative extractable water (REW)	Indicator of soil water availability	Water	Continuous	NA	Yes	No

## Materials and Methods

### Site Characteristics and Plant Material

A mesocosm experiment was set up outdoors in autumn 2012 in Clermont-Ferrand, France (45°46′ N, 03°08′ E, 350 m a.s.l.) under a semi-continental climate (annual mean temperature 12.4°C, mean annual precipitation 579 mm). Five species were selected (four grasses and one legume) from temperate and fertile upland grasslands: *Dactylis glomerata*, *Festuca arundinacea*, *Poa pratensis*, *Trisetum flavescens*, and *T. repens*. *Dactylis* and *Festuca* are tall deep-rooted grass species; *Poa* and *Trisetum* are short shallow-rooted grasses. The unbalanced representation of legume compared to grass is linked to the low abundance of legumes in fertile upland grassland (Louault et al., 2005). Although the root systems of grasses are essentially concentrated in topsoil, a significant part of the roots can also grow deeper than 1 m (Zwicke et al., 2015). *Trifolium* has an intermediary root system pattern with a more even root distribution along the vertical column, and less root density at 20 cm than grasses (Caradus, 1977; Kutschera and Lichtenegger, 1992; Skinner and Comas, 2010).

### Experimental Design

Fifty-three large cylindrical pots (37.5 cm in diameter, 93 cm deep, 100 L) were filled with granitic brown soil (12% clay, 17% loam, 58% sand, 13% organic matter) extracted from an upland grassland (45°43′ N, 03°01′ E, 850 m a.s.l.), sieved (20 mm mesh) and mixed with slow-release fertiliser (3.5 kg m^-3^, NPK 14-7-14 Multicote 12, Haifa, Israel). A 5 cm layer of pozzolan was placed at the bottom of each pot to improve drainage, *via* holes at the bottom. Before being filled with soil, each pot was equipped with a transparent acrylic tube (40 cm in length, 5.5 cm in inside diameter) inserted horizontally at a depth of 80 cm for root observation (see Root Measurements). To reduce soil warming due to light radiation, the pots were insulated with a home-made polystyrene casing (50 mm thick; Styrodur^®^, BASF, France).

In autumn 2012, monocultures, two- and five-species mixtures were established by planting tillers from mature grass plants that had been grown in large containers for 2 years before the experiment (Zwicke et al., 2015), and by sowing *Trifolium* (Merwi variety, medium leaf size). Five types of monoculture (*Dactylis*: dg, *Festuca*: fa, *Poa*: pp, *Trisetum*: tf, *Trifolium*: tr), 10 types of two-species mixtures (*Dactylis*-*Festuca*: dg-fa, *Dactylis*-*Poa*: dg-pp, *Dactylis*-*Trisetum*: dg-tf, *Dactylis*-*Trifolium*: dg-tr, *Festuca*-*Poa*: fa-pp, *Festuca*-*Trisetum*: fa-tf, *Festuca*-*Trifolium*: fa-tr, *Poa*-*Trisetum*: pp-tf, *Poa*-*Trifolium*: pp-tr, *Trisetum*-*Trifolium*: tf-tr) and one five-species mixture (dg-fa-pp-tf-tr) were established each with four and three replicates for monocultures and mixtures, respectively. Each pot initially contained 30 individuals with an equal proportion of species in the mixtures. Five types of sward were considered: monocultures without *Trifolium* (1-, 16 replicates), monocultures with *Trifolium* (1+, 4 replicates), two-species mixtures without (2-, 18 replicates) and with (2+, 12 replicates) *Trifolium*, and five-species mixtures with *Trifolium* (5+, 3 replicates).

### Water Use and Soil Water Content Measurements

From April 2013 (Day of year: DOY 112 year 1) to May 2014 (DOY 132 year 2), 33 of the 53 pots were set on weighing scales (60 cm × 60 cm, Arpege Master K, type N PAC + SAT MB, France) to continuously measure the actual evapotranspiration (ET, kg) of the plant canopy by the daily changes in pot weight (Zwicke et al., 2015). Due to a limited number of scales, ET measurements were performed on 11 pots of monocultures, 20 pots of two-species mixtures, and 2 pots of five-species mixture. A correction of daily ET was applied to allow for weight change due to rain or irrigation events. Throughout the experimentation (20 months), all the pots were maintained at 80% of field capacity by watering or rainfall events.

Soil water content (SWC) was assessed using two methods, by gravimetry (daily change in pot gravimetric soil moisture), and by direct measurement with soil probes. Gravimetric SWC was expressed as daily soil relative extractable water (REW*_t_*) calculated as:

$${REW}_{t}=\frac{{soil\,\;moisture}_{t}-{soil\,\;moisture}_{\min}}{{soil\,\;moisture}_{\max}-{soil\,\;moisture}_{\min}},$$

where soil moisture_t_, soil moisture_min_ and soil moisture_max_ are respectively the current, minimum and maximum gravimetric soil moistures measured at time t, in drought (min value = 0.054) and well-watered (max value = 0.379) conditions. The minimum value was obtained from a parallel study done with similar soil and pots.

Sixteen pots (5 monocultures, 10 two-species mixtures, 1 five-species mixture) were also equipped with SWC sensors (ECHO-5, Decagon, USA) inserted horizontally at three depths (15, 30, 50 cm) and connected to a datalogger (EM50, Decagon, USA). From April 2013 (DOY 112 year 1) to June 2014 (DOY 133 year 2), SWC (m^3^ m^-3^) was measured every 30 min, and data were averaged at daily scale.

### Above-Ground Biomass and Water-Use Efficiency

Vegetation was cut to 5 cm height at seven dates between April 2013 and June 2014, corresponding to five and two cuts in 2013 and 2014, respectively, to mimic current mowing practice for such vegetation. The first cut in April corresponded to a standardized harvest. Thus six cutting dates were used to define different periods of vegetation biomass production (g pot^-1^) during the experiment: spring year 1 (DOY 113 to 143), early summer year 1 (DOY 144 to 190), late summer year 1 (DOY 191 to 224), autumn year 1 (DOY 225 to 280), autumn year 1 – early spring year 2 (DOY 281 to 101) and spring year 2 (DOY 102 to 161). At each cut, plant material was sorted by species, and green leaves were separated from inflorescences. Above-ground biomass, comprising all organs, was determined after oven-drying (60°C for 48 h) and weighing. Before the spring cut in year 1 and the spring cut in year 2, photosynthetic active radiation (PAR) extinction was measured using a Sunfleck ceptometer (Decagon Devices, Inc., Pullman, WA, USA) to delimit two horizontal canopy layers (top and bottom), each contributing to approximately 50% of the absorbed PAR. For a given species and mixture, percentage of biomass of species present in the top layer (Biom st1, %) was calculated as the ratio of Biom st1 to total biomass.

For each year of measurement, integrated water-use efficiency (WUE, g kg^-1^) was calculated as the ratio of annual above-ground biomass to annual evapotranspiration (sum of daily evapotranspiration).

### Leaf Traits and N Measurements

Specific leaf area (SLA, m^2^ kg^-1^) and leaf dry matter content (LDMC, mg g^-1^) were measured for all pots and species, using two leaves per species per pot, at two sampling dates (spring year 1: DOY 136 and year 2: DOY 157) to characterize species’ strategies for resource acquisition and resource use. Above-ground biomass and SLA were used to calculate community leaf area (L.area, m^2^ pot^-1^) for each period. Plant vegetative height of each species present in each community was measured throughout the experiment by averaging five measurement points per species and per pot (measurements at 18 dates between spring year 1 and spring year 2). Increase in height between two consecutive dates divided by day number was calculated and defined as height growth rate. Maximum values for each period of cuts was calculated and averaged by treatment (H.growth, cm day^-1^). Green leaves sampled at each cutting date from May 2013 to June 2014 were oven-dried (60°C, 48 h) and ball-milled (MM200, Retsch, Germany). Samples weighing 1 mg were combusted and analyzed for leaf nitrogen content (N, %; IsotopeCube, Elementar, Hanau, Germany) and leaf ^13^C isotopic composition (Isoprime 100, IsoPrime, Manchester, UK) at the stable isotope facility at INRA Nancy, France. Carbon isotopic composition (δ^13^C, aaa) was expressed with an analytical precision of 0.2aaa; (standard deviation) and measured for three periods of biomass production (spring year 1, autumn year 1, and spring year 2).

Nitrogen yield (Nyield, gN pot^-1^) was calculated by multiplying N by biomass, and Nyield/ET (g N kg^-1^) was the ratio of Nyield to evapotranspiration. This trait expresses the link between N and water use and thus the cost of water necessary to produce shoot N.

### Root Measurements

From spring 2013 to May 2014, root images were recorded each month or twice a month using a minirhizotron system (BTC-2, Bartz Technology, USA) inserted into the acrylic tubes toward the base of each pot. At each date, 11 images (each 1.35 cm × 1.8 cm) were recorded, and length of root segments was measured manually using WinRHIZOTronMF software (V2005a, Regent Instruments, Canada). For each date, length was modified according to growth event, and was expressed per unit tube area (mm cm^-2^). For each tube and date, the root length of the 11 images was averaged. Increase in root length between two consecutive dates divided by day number was calculated and defined as root length growth rate. Maximum values for each period of cuts was calculated and averaged by treatment (R.growth, mm cm^-2^ day^-1^). During summer of year 2, two soil cores per pot (3 cm diameter, 20 cm depth) were collected in June 2014 (DOY 153 year 2). For each pot, the two cores were mixed and the roots were washed, oven-dried (60°C, 48 h) and weighed, and dry mass expressed per soil volume (R.mass, mg cm^-3^).

### Community-Weighted Mean Traits

For each mixture, community-weighted mean (CWM) traits were calculated on 10 variables (Biom st1, δ^13^C, H.growth, L.area, LDMC, N, Nyield/ET, REW, R.growth, WUE). The following equation was used: $CWM=\Sigma_{i=1}^{S}P_{i}t_{i},$ where *S* is the number of species in the community and *t_i_* are species-specific trait values; *p_i_* are the species proportion (i) in total biomass for LDMC, (ii) in green leaves for N, and (iii) in leaf area for δ^13^C. Other traits were obtained directly at the community level: R.growth, Biom st1, L.area, WUE, Nyield/ET and REW. These 10 variables were previously selected for their potential function in link with N, water and/or light resources (**Table 1**) and then pairwise-tested to bring out the absence of correlation.

Functional trait diversity was computed as Raò’s quadratic entropy (FD_Q_, Leps et al., 2006) on six of the 10 variables because they were measured at species level (Biom st1, δ^13^C, H.growth, L.area, LDMC, N), whereas the others were measured at community level (Nyield/ET, REW, R.growth, WUE). All information concerning the calculation and analysis of FD_Q_ traits are explored in Supplementary Material.

### Diversity Effects

Above-ground biomass for each period was used to calculate the net diversity effect, which is the difference, summed across species, between observed and expected biomass in mixtures. The expected biomass of each species in a mixture is the product of biomass in monoculture and its proportion in total above-ground biomass in the mixture. We used the method described by Loreau and Hector (2001) to additively partition the net diversity effect in mixtures into complementarity and selection effects. Positive complementarity effect occurs if species yields in a mixture are on average higher than expected on the basis of the weighted average monoculture yield of the component species. We additionally calculated the proportional deviation of species *i*’s biomass from its expected value (D*_i_*), which reveals the sign and magnitude of the net effect on each species of the interactions with the other species in a mixture (Loreau, 1998), according to the equation

$$D_{i}=\frac{{biomass}_{{obs}_{i}}-{biomass}_{\exp_{i}}}{{biomass}_{\exp_{i}}},$$

biomass_obsi_ and biomass_exp_*_i_* are the measured and expected biomasses of species *i*, respectively. Similarly, *D*_i_ was calculated for averaged grasses (D_Grass_) and *Trifolium* (D_Leg_) species. Positive values for D_Grass_ or D_Leg_ show that grasses or *Trifolium* produced more biomass in the mixture than expected based on monoculture, suggesting higher intra- than interspecific competition, facilitation or niche complementarity between species. In contrast, a negative D_Grass_ or D_Leg_ indicates that grasses or *Trifolium* produced less biomass in mixture than expected, suggesting a higher inter than intra-specific competition.

### Statistical Analyses

A nested linear model was used to test the effect of species-richness (S: 1, 2) and *Trifolium* presence in the monocultures and two-species mixtures (Leg: leg-, leg+) on above-ground biomass, ET, WUE, L.area, N, Nyield/ET, REW, deep R.growth and topsoil root mass. Using species composition as nested random factor, we tested the effects of species richness, legume presence and their interaction. Data were first transformed when necessary (square root or boxcox transformation) to conform to the assumptions of normality and homogeneity of variances (R package car). Analysis of variance (ANOVA) on mixed effect models and the *post hoc* Tukey test were performed for each annual data, whole experiment and for six periods in case of biomass (R packages lme4 and lsmeans).

Then, two nested linear mixed models were used to test the effect of species-richness (S: 1, 2, 5 species, model 1) and *Trifolium* presence in the two-species mixtures (Leg: leg-, leg+, model 2) on above-ground biomass and the same set of traits (ET, WUE, L.area, N, Nyield/ET, REW, deep R.growth and topsoil root mass). For model 1, we tested the effects of species richness (diversity effect) as fixed factor and species composition as random factor nested within species richness in order to assess contributions from species identity and richness by partitioning the variance between identity nested under richness (Giller et al., 2004; Vanelslander et al., 2009). However, it was not possible to separate this effect for the five-species mixture because it was not truly replicated like other sward. Although this is a drawback in the diversity effect analysis, we included this mixture because it supplies information on plant interactions when all species are grown together (Vanelslander et al., 2009). For model 2, we tested the effects of legume presence in the two-species mixtures as fixed factor and species composition as random factor nested within legume presence. ANOVA on mixed models (models 1 and 2) were performed for each annual data and whole experiment.

Effects of S (1, 2, 5) and Leg were tested using nested repeated measures ANOVA on REW and SWC at three depths, with the fixed factors of models 1 and 2 and with species composition, pot and date as random factors (R packages nlme and lsmeans).

Standardized principal components analyses (PCA) were performed to explore relationships between sward types (monocultures and mixtures) and 10 variables including traits related to light, nitrogen and water uses averaged over the experimental period (R package ade4).

Given the observed importance of CWM predictors over FD traits explaining variation in above-ground biomass production and diversity effects (Supplementary material), we used statistical models with the CWM of 10 variables in order to select the main traits contributing to the amount of explained variation in above-ground biomass production and diversity effects. Within each class of models, we selected the best fit based on leave-one-out cross validation (R packages car and leaps). The coefficient of determination *R*^2^ is given as a summary measure for explained variation. The final selected models contain five traits, as adding additional variables did not significantly increase *R*^2^. We also studied the relative importance of each variable within each model selected using the proportional marginal variance decomposition metric proposed by Feldman (2005), which can be interpreted as a weighted average over orderings among regressors, with data-dependent weights (R package relaimpo). The qualitative exclusion/inclusion of traits has recently been generalized to a more quantitative approach where relative weights for the different traits can additionally be estimated (Schumacher and Roscher, 2009). For PCAs and model selection, average data for the whole experiment and summer of year 2 (period 102–161) are shown, owing to a more pronounced effect of legume presence at the end of the experiment. All statistical analyses were carried out with R software (R Core Team, 2009).

## Results

### Species Richness Effect

**FIGURE 1:**
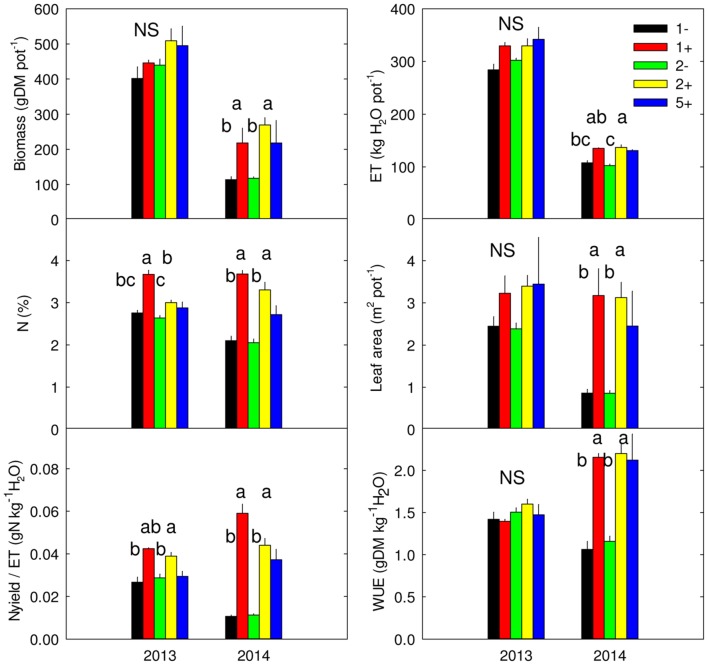
**Above-ground biomass, evapotranspiration (ET), community-weighted mean of N (N), leaf area (L.area), water-use efficiency (WUE) and ratio of Nyield to ET (Nyield/ET) measured in 2013 and 2014, for monocultures without (1-) or with *Trifolium repens* (1+), two-species mixtures without (2-) and with (2+) *T. repens* and five-species mixture with *T. repens* (5+).** Mean values + SEM are shown. For each variable and year, *post hoc* test was performed for all treatments except 5+; different letters correspond to statistical differences (*P* ≤ 0.05). NS: *P* > 0.05.

**FIGURE 2:**
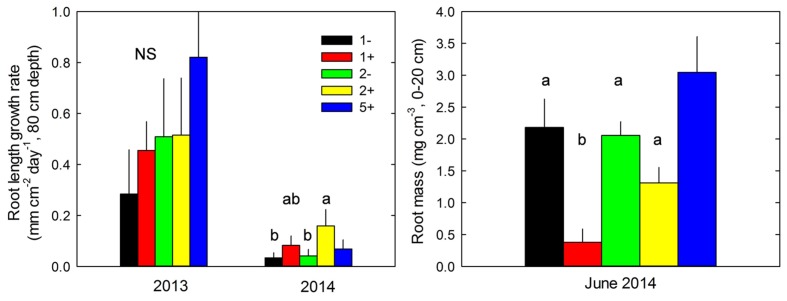
**Maximum root length growth rate measured at 80 cm depth in 2013 and 2014 and root mass measured at 20 cm in June 2014, for monocultures without (1-) or with *T. repens* (1+), two-species mixtures without (2-) and with (2+) *T. repens* and five-species mixture with *T. repens* (5+).** Mean values + SEM are shown. For each variable and year, *post hoc* test was performed for all treatments except 5+; different letters correspond to statistical differences (*P* ≤ 0.05).

**FIGURE 3:**
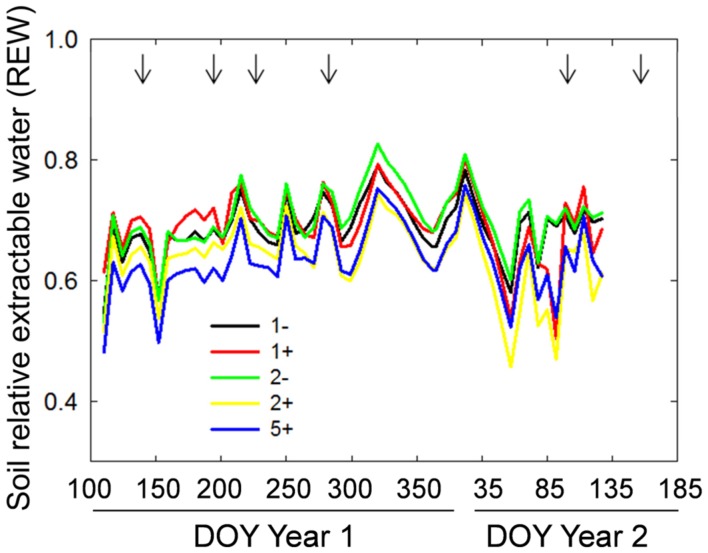
**Temporal dynamics of soil relative extractable water (REW) for monocultures without (1-) or with *T. repens* (1+), two-species mixtures without (2-) and with (2+) *T. repens* and five-species mixture with *T. repens* (5+).** Seven-days averages are shown. Arrows correspond to cutting events.

Species richness (S: 1, 2, 5) had no significant effect on seven of the eight plant variables including above-ground biomass (Model 1 in **Table 2**; **Figures 1** and **2**). The only exception was observed during the first year for the maximum deep root length growth rate which had 2.4-fold higher values in the five-species mixtures compared to monocultures (**Figure 2**). In addition in summer of the second year soil REW had 10% lower values in the five-species mixtures compared to monocultures (*P* = 0.087 and *P* = 0.068, **Figure 3**; **Table 3**). This trend was also observable in SWC at 15 and 50 cm (Supplementary Figure S1). We also observed a trend toward positive net diversity and complementarity effects in five-species mixtures especially in summer of year 2 (40.3 g pot^-1^ and 38 g pot^-1^, respectively, data not shown). Similarly, species richness (S: 1, 2) had no significant effect on five of the eight plant variables (**Table 4**). Indeed, doubling the number of species (1 vs. 2) only had positive effect on above-ground biomass, Nyield/ET and R.growth in year 2, whereas doubling the number of grass species in the sward had no effect on plant or soil characteristics (1- vs. 2-, **Figures 1–3**). Otherwise, we measured a strong Leg effect on the eight plant variables in monocultures and two-species mixtures, mostly in year 2 (*P* < 0.001; **Table 4**). Then, no significant interaction between S and Leg was observed, except for topsoil root mass (R.mass) in year 2, meaning that legume presence effect was independent of species richness (**Figures 1** and **2**).

**Table 2:** Effects of species richness (S, Model 1), *Trifolium repens* presence in two-species mixtures (Leg, Model 2) on above-ground biomass, evapotranspiration (ET), leaf area (L.area), community-weighted mean of N (N), plant water-use efficiency (WUE), ratio of Nyield to ET (Nyield/ET), maximum root length growth rate measured at 80 cm depth (R.growth), and root mass measured at 20 cm (R.mass).

		Model 1		Model 2	
Variables	Period	Num/Den DF	S *P*-value	Num/Den DF	Leg *P*-value
Biomass	2013	2/14.1	0.336	1/8	0.206
	2014	2/14.1	0.247	1/8	**<0.001**
	Total	2/14.1	0.226	1/8	**0.003**
ET	2013	2/14.1	0.148	1/8	0.107
	2014	2/14	0.422	1/8	**<0.001**
	Total	2/14.1	0.145	1/8	**<0.005**
L.area	2013	2/14.1	0.312	1/8	**0.020**
	2014	2/14.1	0.283	1/8	**<0.001**
	Total	2/14.0	0.251	1/8	**<0.001**
*N*	2013	2/14.1	0.781	1/8	**0.016**
	2014	2/14.0	0.593	1/8	**<0.001**
	Total	2/14.0	1	1/8	**<0.002**
WUE	2013	2/14.0	0.754	1/8	0.404
	2014	2/14.0	0.144	1/8	**<0.001**
	Total	2/14.1	0.307	1/8	**0.001**
Nyield/ET	2013	2/14.2	1	1/8	**0.015**
	2014	2/14.0	0.168	1/8	**<0.001**
	Total	2/14.1	0.734	1/8	**<0.001**
R.growth	2013	2/14.2	**<0.001**	1/8	0.176
	2014	2/13.9	0.122	1/8	**<0.001**
	Total	2/14.2	**<0.001**	1/8	0.083
R.mass	2014	2/14.7	0.181	1/8	**0.049**

*Averages or cumulated values over the whole experiment period were used for all variables, except for root mass, for which data of June 2014 were used. Bold and underlined P-values correspond to significant differences: P < 0.05 and P < 0.10, respectively.*

*For S: 1, 2, and 5 species treatments were compared; for Leg: presence (+) or absence (-) of T. repens species in the two-species mixtures were compared; NumDF and DenDF are degrees of freedom of term and degrees of freedom of error term (which can be fractional in residual maximum likelihood analysis).*

**Table 3:** Effects of species richness (S, Model 1) and *T. repens* presence in two-species mixtures (Leg, Model 2) on soil relative extractable water (REW).

		Model 1		Model 2	
Year	Period	Num DF	S *P*-value	Num DF	Leg *P*-value
2013	113–143	2	0.323	1	0.258
2013	144–190	2	0.087	1	0.280
2013	191–224	2	0.068	1	0.165
2013	225–280	2	0.143	1	0.088
2013–2014	281–101	2	0.396	1	**0.007**
2014	102–132	2	0.200	1	**0.003**

*Averages values over each period were used. P-values are shown. Bold and underlined values correspond to significant differences: P < 0.05 and P < 0.10, respectively.*

*For S: 1, 2, and 5 species treatments were compared; for Leg: presence (+) or absence (-) of T. repens species in two-species mixtures were compared; NumDF is degrees of freedom of term.*

**Table 4:** Effects of species richness (S: 1, 2), *T. repens* presence in one and two-species mixtures (Leg) and their interaction on above-ground biomass, evapotranspiration (ET), leaf area (L.area), community-weighted mean of N (N), plant water-use efficiency (WUE), ratio of Nyield to ET (Nyield/ET), maximum root length growth rate measured at 80 cm depth (R.growth), and root mass measured at 20 cm (R.mass).

Variables	Period	Num/Den DF	S *P*-value	Leg *P*-value	S x Leg *P*-value
Biomass	2013	1/11	0.356	0.291	0.791
	2014	1/11	**0.043**	**0.0001**	0.594
	Total	1/11	0.157	**0.013**	0.663
ET	2013	1/11	0.332	0.095	0.765
	2014	1/11	0.800	**0.001**	0.674
	Total	1/11	0.245	**0.008**	0.704
L.area	2013	1/11	0.626	**0.038**	0.825
	2014	1/11	0.111	**<0.0001**	0.879
	Total	1/11	0.312	**<0.001**	0.847
*N*	2013	1/11	0.654	**0.031**	0.759
	2014	1/11	0.083	**<0.0001**	0.944
	Total	1/11	0.622	**0.001**	0.220
WUE	2013	1/11	0.430	0.675	0.589
	2014	1/11	0.076	**<0.0001**	0.840
	Total	1/11	0.198	**0.012**	0.571
Nyield / ET	2013	1/11	0.647	**0.013**	0.636
	2014	1/11	**0.007**	**<0.0001**	0.090
	Total	1/11	0.392	**0.0001**	0.252
R.growth	2013	1/11	0.190	0.570	0.639
	2014	1/11	**0.011**	**<0.0001**	0.148
	Total	1/11	0.150	0.350	0.738
R.mass	2014	1/11	0.392	**0.001**	**0.020**

*Averages or cumulated values over the whole experiment period were used for all variables, except for root mass, for which data of June 2014 were used. Bold and underlined P-values correspond to significant differences: P < 0.05 and P < 0.10, respectively.*

*NumDF and DenDF are degrees of freedom of term and degrees of freedom of error term.*

### Effect of *Trifolium* Presence in Two-Species Mixtures on Community Traits

*Trifolium* presence had a significant effect in the second year on all plant characteristics recorded (Model 2 in **Table 2**; **Figures 1** and **2**). From autumn of year 1 (DOY 280, **Tables 2** and **3**), biomass, ET, L.area, N, WUE, and Nyield/ET increased significantly in sward containing *Trifolium* species, which corresponded to its higher proportion in the biomass (**Table 5**). In addition, at the end of the experiment, N content of grasses growing with *Trifolium* was 53% higher than that of grasses growing without it (Supplementary Table S1). In the presence of *Trifolium*, REW measured from autumn of year 1 decreased (**Figure 3**; **Table 3**) whereas deep root growth (**Figure 2**) increased. Data of REW measured at whole soil water availability were confirmed with measurement of SWC at 50 cm (-14%, *P* < 0.01 from DOY 280; Supplementary Figure S1).

**Table 5:** Above-ground biomass (g pot^-1^) and *T. repens* proportion in the biomass for each cut in 2013 and 2014 and cumulated for the whole experiment.

Year		2013				2014		Whole experiment
Variable	Treat.	113–143	144–190	191–224	225–280	281–101	102–161	113 y1–161 y2
Biomass	1-	92.6 a	149.6 a	85.3 a	74.1 c	59.1 c	54.6 b	515.2 b
	1+	31.2 b	159.7 a	120.4 a	134.8 ab	100.3 ab	118.3 a	664.6 ab
	2-	90.5 a	176.8 a	98.0 a	74.1 c	56.7 c	60.7 b	556.6 b
	2+	84.4 a	176.8 a	115.3 a	132.4 a	114.3 a	155.1 a	778.2 a
	5+	93.5	190.7	121.6	89.4	67.6	150.4	713.1
*T. repens*	2+	0.028 a	0.219 a	0.356 a	0.520 a	0.617 a	0.680 a	0.433 a
Proportion	5+	0.011 a	0.011 b	0.037 b	0.107 b	0.337 a	0.433 a	0.170 a

*Average values for five (1-, 1+, 2-, 2+, 5+) and two (2+, 5+) diversity treatments (Treat.) are shown. For a given period, post hoc tests were performed to compare biomass of all treatments except 5+ and to compare T. repens proportion between 2+ and 5+ treatments; different letters indicate statistical difference between treatments (P ≤ 0.05).*

### Time Dependence of Diversity Effects in Two-Species Mixtures with *Trifolium*

**FIGURE 4:**
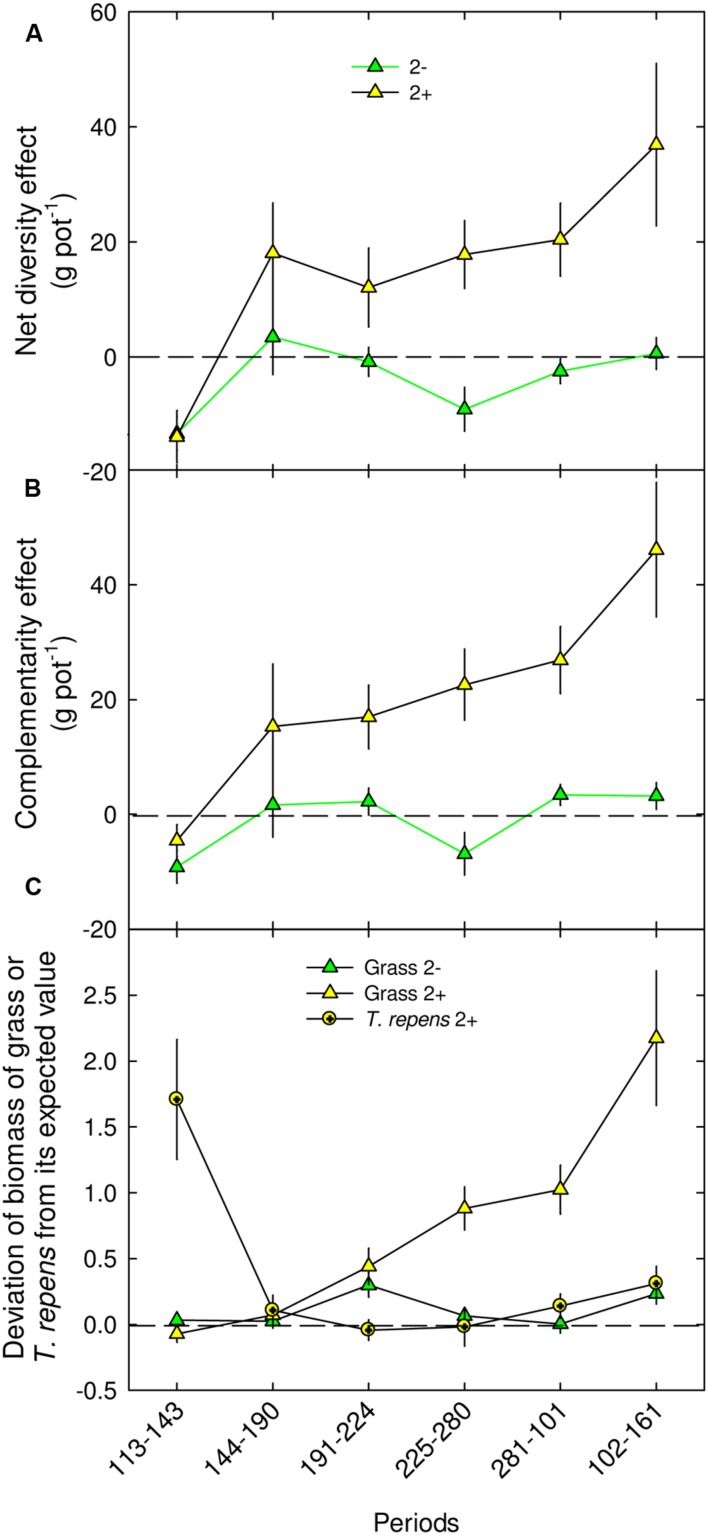
**Net diversity effect (A), complementarity effect (B) and proportional deviation of grass and *T. repens* species biomass from its expected value (C) in mixtures with two species: 2-, 2+, for different periods in 2013 and 2014.** Mean values ± SEM are shown. For each period and sward type, different letters correspond to statistical differences (*P* ≤ 0.05).

A significant net positive diversity effect was observed on two-species mixtures, but only with *Trifolium* presence (2+), from autumn in year 1 (+17.8 g pot^-1^; *P* < 0.01, DOY 225–280) to spring in year 2 (+36.9 g pot^-1^; *P* < 0.01, DOY 102–161; **Figure 4A**). This over-yielding was mostly due to a positive complementarity effect (75%, +22.6 and 46.1 g pot^-1^, for DOY 191–224 until DOY 101–161, respectively; **Figure 4B**), the selection effect being nil or negative throughout the experiment (data not shown). Also, the net diversity effect measured on above-ground biomass in the two-species mixture with *Trifolium* can be sorted into two components, D_Grass_ and *D*_Leg_ (**Figure 4C**). D_Grass_ was significantly positive from summer in the first year (*P* < 0.01, DOY 191–224) until the following spring (*P* < 0.001, DOY 101–161), while D_Leg_ was significantly positive during spring in the first year (*P* < 0.001, DOY 113–143) and in the second year (*P* < 0.05, DOY 102–161; **Figure 4C**). In addition, for the two-species mixtures, D_Grass_ measured with *Trifolium* (2+) was higher than the D_Grass_ measured without it (2-) from autumn ear 1 to the following spring (*P* < 0.001, DOY 225–280, **Figure 4C**), highlighting a significant effect of the legume species on grass biomass in mixtures.

### Trait Syndromes of Monocultures and Mixtures

**FIGURE 5:**
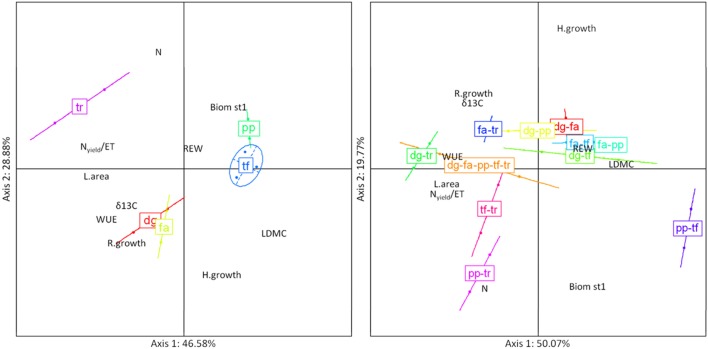
**Standardized principal component analysis (PCA) combining 10 traits for the five-species monocultures (left) and community-weighted means of trait values (CWM) on 10 functional traits for mixtures of two and five species-mixtures (right), averaged over the experimental period.** Biom st1, percentage of biomass in the top canopy layer (%); δ^13^C, leaf C isotopic composition (aaa); H.growth, growth height (cm day^-1^); L.area, leaf area (m^2^ pot^-1^); LDMC, leaf dry matter content (mg g^-1^); N, N community-weighted mean (%); Nyield/ET, ratio of Nyield to evapotranspiration (g kg^-1^); REW, soil relative extractable water; R.growth, max root length growth rate at 80 cm depth (mm cm^-2^ day^-1^); WUE, integrated water use efficiency (g kg^-1^). dg, fa, pp, tf, tr correspond to monocultures of *Dactylis, Festuca*, *Poa*, *Trisetum* and *Trifolium*, dg-fa, dg-pp, dg-tf, dg-tr, fa-pp, fa-tf, fa-tr, pp-tf, pp-tr and tf-tr correspond to two-species mixtures of *Dactylis*-*Festuca*, *Dactylis*-*Poa*, *Dactylis*-*Trisetum*, *Dactylis*-*Trifolium*, *Festuca*-*Poa*, *Festuca*-*Trisetum*, *Festuca*-*Trifolium*, *Poa*-*Trisetum*, *Poa*-*Trifolium*, *Trisetum*-*Trifolium*; dg-fa-pp-tf-tr corresponds to the five-species mixture *Dactylis*-*Festuca*-*Poa*-*Trisetum*-*Trifolium*.

For the PCA based on 10 plant traits of monocultures, the two axes explained 75.5% of variation (**Figure 5**, left). The first axis, accounting for 46.6% of species variation in multiple traits, had a high positive loading for LDMC together with high negative loadings for Nyield/ET, L.area, and WUE. The first principal component clearly separated short grasses (*Poa*, *Trisetum*) from *Trifolium*. The second principal component accounted for about 28.9% of species variation in multiple traits. This axis had a high positive loading for N and high negative loadings for H.growth and R.growth. The second principal component clearly separated short grasses (*Poa*, *Trisetum*) and the legume (*Trifolium*) from tall grasses (*Dactylis*, *Festuca*).

For the mixtures, the two axes explained 69.8% of variation (**Figure 5**, right). The first principal component accounted for 50.1% of variation among mixtures. This axis had a high positive loading for LDMC and high negative loadings for L.area, Nyield/ET and WUE. The first axis clearly separated grass mixtures from mixtures with *Trifolium*. The second axis explained about 19.8% of variation in traits among mixtures, and was characterized by a high positive loading for H.growth and high negative loadings for N and Biom st1.

### Prediction of Biomass Production, Net Diversity, and Complementarity Effects with Community Traits

For the scale of the whole experimental period (20 months), final prediction models containing five traits yielded high prediction of biomass (*R*^2^ = 0.950), net diversity (*R*^2^ = 0.932) and complementarity (*R*^2^ = 0.927; **Table 6**). Biomass production was best explained by the combination of WUE, L.area, REW, R.growth and H.growth (*R*^2^ = 0.950, **Table 6**). The main contributors were WUE (49.3%) and L.area (41.4%), ordering species along strategy spectra with regard to water acquisition and use, more than N or light capture. Indeed, leaf area was mostly used as a proxy of evapotranspiration considering the strong correlation measured with ET for the whole experiment (*R*^2^ = 0.58, *P* < 0.0001) as well as in summer of year 2 (*R*^2^ = 0.50, *P* = 0.003). For the net diversity effect, a combination of CWM traits characterizing efforts in water and N acquisition and use were selected: L.area (35.6%), WUE (24.5%), and N (24.2%). The best prediction of complementarity effect then included the same variables: L.area (39.8%), WUE (27.2%), N (15.6%), and also Nyield/ET (10.4%) and H.growth (6.9%). These ordered mixtures mainly along strategy spectra with regard to water acquisition and use, but also N use. Similarly, taking into account only the summer period of the second year, L.area a trait mainly correlated to water use, principally explained biomass (97.5%), net diversity (89.8%) and complementarity (76.3%) effects (**Table 6**).

**Table 6:** Summary of best statistical models based on 10 community-weighted means of trait values (CWM) predictor variables for community biomass, net diversity effect, and complementarity effect.

Period		*R*^2^	10 traits: CWM					
Whole experiment	Biomass	**0.950**	Selected traits	WUE	L.area	REW	R.growth	H.growth
			% R^2^	49.3	41.4	3.4	3.1	2.8
	Net diversity	**0.932**	Selected traits	L.area	WUE	N	Nyield/ET	H.growth
	Effect		% R^2^	35.6	24.5	24.2	9.8	6.1
	Complementarity	**0.927**	Selected traits	L.area	WUE	N	Nyield/ET	H.growth
	Effect		% R^2^	39.8	27.2	15.6	10.4	6.9

Summer year 2	Biomass	**0.968**	Selected traits	L.area	LDMC	REW	Biom st1	Nyield/ET
			% R^2^	97.5	0.9	0.8	0.8	0.7
	Net diversity	**0.929**	Selected traits	L.area	LDMC	WUE	Biom st1	REW
	Effect		% R^2^	89.8	5.1	4.0	1.0	0.2
	Complementarity	**0.883**	Selected traits	L.area	R.growth	Biom st1	LDMC	WUE
	Effect		% R^2^	76.3	7.4	6.2	5.4	4.6

*The final selected models contain five traits, as adding additional variables did not significantly increase R. The five selected traits and relative importance of R^2^ (% R^2^) are shown for whole experiment and summer of year 2 (period 102–161). Models were developed with both two and five-species mixtures.*

## Discussion

Overall, our results confirmed that functional diversity through *Trifolium* presence is more important than species richness to explain biomass production and diversity effects (Tilman et al., 2002). Our data showed no significant effect of species richness (1, 2, 5 species) for biomass production and traits related to N and water-use. In fact the absence of richness effect contradicts with most of diversity experiments (Loreau and Hector, 2001; Spehn et al., 2005) and should be due to the lack of statistical power necessary to test species richness. Moreover, without taking into account the five-species mixtures, a supplementary test of richness effect made on monoculture and two-species mixtures underlines that species richness had minor effect relative to that of *Trifolium* presence on most of the traits including biomass (**Table 4**). Thus, species richness effects in two-species mixtures seem to be confounded with the presence of *Trifolium*. This result appears consistent with most of diversity experiments, which showed positive diversity effect on biomass production in mixtures including legume species (Loreau and Hector, 2001; Spehn et al., 2005). Then in our experiment, by using a set of traits related to light, N and water uptake and use, we showed that both the two and five-mixtures with *Trifolium* exhibited similar trait syndromes and thus similar strategies for resources uptake and use (**Figure 5**). Together with trends toward similar biomass production, diversity and complementarity effects in the two and five-mixtures with *Trifolium*, these results highlighted that the lack of significant effect in the five-mixtures should be due to the slow development of *Trifolium* (**Table 5**). Altogether our results confirmed that the presence of *Trifolium* promoted biomass production, over-yielding and had a pronounced effect on traits related to N and water use, especially in the two species mixtures. This emphasised that *Trifolium* presence is the main determinant of above-ground production and of diversity effects in our mixtures whatever the grass species associated.

According to others studies (Loreau and Hector, 2001; Spehn et al., 2005; Cardinale et al., 2007), complementarity effects mainly explained the net diversity effects we observed in the two species mixtures. Facilitation and niche differentiation, both included in the complementarity effect described by Loreau and Hector (2001), have been suggested as potential underlying mechanisms for positive diversity effects on biomass production. Higher shoot nitrogen measured in sward containing *Trifolium* from the beginning of the experiment and in the associated grass at the end of the experiment underline N facilitation induced by the legume (Spehn et al., 2002; Hille Ris Lambers et al., 2004; Temperton et al., 2007; Brooker et al., 2008; Marquard et al., 2009). Low root mass at the shallow soil layer in mixtures containing *Trifolium*, as well as positive D_grass_, also suggest N facilitation by the legume in summer of year 2. Furthermore, the analysis based on model selection highlighted that traits related to N acquisition and use (N, Nyield/ET) were important determinants of net diversity and complementarity effects, but not of biomass production. Unexpectedly for the second year, N traits disappeared from the selected models although N facilitation induced by *Trifolium* was observed. Indeed the selected models highlighted the importance of leaf area, a trait highly correlated with evapotranspiration in our experiment, which suggest that traits related to water use mostly explained biomass, diversity and complementarity effects in mixtures. Furthermore, traits related to above-ground dominance (light resource) were also included in the models for the whole experiment, but showed less than 7% of the total variance and disappeared in the model developed for summer of the second year. Thus, light availability appears to play a minor role for the outcome of plant–plant interactions including *Trifolium* (but see FD traits related to light in the Supplementary Material). Overall model selection underlined the importance of water compared to N traits as predictors of biomass production and diversity effects. However, we cannot rule out that leaf area which is an integrated trait can be related to N and light capture.

Our results suggest that resources other than N should also be considered to explain the positive effects of legumes in grass-legume mixtures (Hoekstra et al., 2014). Using direct measurement of evapotranspiration and soil REW, our data highlighted that the presence of *Trifolium* in mixtures promoted water use, suggesting a more exhaustive exploitation of soil water due to higher complementarity (Høgh-Jensen and Schjoerring, 1997; Verheyen et al., 2008). Although higher water use due to niche complementarity effect was proposed as underlying mechanism of diversity-productivity relationship, few studies measured water use in diverse mixtures and inconsistent results have been observed. Some authors showed that in more species rich mixtures soil moisture either decline as a result of higher transpiration and biomass (De Boeck et al., 2006; Mokany et al., 2008; Verheyen et al., 2008), increased which was attributable to the increased shading by the canopy in more diverse plant mixtures (Rosenkranz et al., 2012), or caused no change (Stocker et al., 1999; Spehn et al., 2000; Leimer et al., 2014). In our experiment, in addition to higher ET, we highlighted both higher root growth and lower SWC in deep soil layer for mixtures containing *Trifolium*. Moreover, in shallow soil layers, low root density of *Trifolium* (Skinner and Comas, 2010) can lead to low competition for water uptake and thus to an increase of soil water availability for the associated grass species, in addition with facilitative effect on soil N induced by *Trifolium*. The model selection analysis also puts into evidence the importance of resources access from deep soil layer through deep root growth rate as a predictor of complementarity effect. This is in line with the findings of several authors who showed that root depth distributions in mixtures were more than twice higher as expected from monocultures (Skinner et al., 2006; Mueller et al., 2013). Moreover, spatial complementarity of the root systems of grass species, by assembling shallow (*Poa*, *Trisetum*) and deep (*Dactylis*, *Festuca*) rooted species, did not lead to higher biomass production or diversity effect. This suggests that vertical root differentiation and an increased deep exploitation of soil resources by the species in grass-*Trifolium* mixtures is due to root plasticity more than inherent different rooting distribution (Mommer et al., 2010; Skinner and Comas, 2010; Mueller et al., 2013).

In artificially manipulated mixtures, many studies underlined the time dependence of diversity effects that could be linked with the duration of the experiment (Cardinale et al., 2007). In case of *Trifolium*-grass mixtures, it is known that legume proportion fluctuates both from year to year and within single growth periods (Frame, 1986). These fluctuations can be linked with effects of abiotic and biotic factors on N_2_ fixation (Soussana and Tallec, 2010). Our data suggest that increase proportion of *Trifolium* in mixtures until the summer in year 2 is associated with its establishment and then with the increase of diversity and complementarity effects. According to Haynes (1980), competition for light induced by grasses, together with competition for shallow soil resources, can slow the development of *Trifolium*. However, consecutive cut events would have decreased the negative shading effect of grass species, leading to progressive aerial establishment of *Trifolium*, which finally capped at about 60% of the above-ground biomass for the two-species mixtures in the year 2. The slow dynamic of *Trifolium* establishment, associated with the progressive N exportation through cuts and thus the likelihood of decrease in N soil content, is coherent with the time dependence of the positive effects of *Trifolium* on biomass production and complementarity. The observation of a time lag for the positive *Trifolium* effects establishment, measured only from autumn in year 1, is also consistent with the results of Spehn et al. (2005), Cardinale et al. (2007), and Reich et al. (2012). Higher deep root growth, lower soil REW and SWC at 50 cm indicate that competition for water induced by grasses having dense, and deep root systems (Zwicke et al., 2015) was sharper in the mixtures than in monocultures. This more stressful condition may have curbed the development of *Trifolium*, known to be drought-sensitive (Grieu et al., 2001). Nonetheless, a strong positive effect of *Trifolium* was measured on the grass species associated and on the community even in case of low proportion of legume in the mixture (30% in the two-species mixtures). Despite it is consistent with Suter et al. (2015), our findings partly conflict with their results which demonstrated a considerable N yield increase with increasing legume proportion up to about 30%, until a plateau is reached. It indicates that almost all of the maximum benefits to N yield from mixing grasses and *T. repens* can be achieved with a modest legume proportion in the mixture. However, in our study we measured a significant correlation between *Trifolium* proportion (and/or *Trifolium* biomass) and D_Grass_, shoot N of the grass associated, net diversity and complementarity effects in mixtures over a wide range of legume proportions. Furthermore, for D_Grass_, we measured the strongest increase when *Trifolium* proportion was superior to 50%. Finally, our results unexpectedly highlighted a mutual facilitative interaction between grass and *Trifolium* in mixtures. Indeed, if our results showed a positive and delayed effect of *Trifolium* on the associated grass species, we also underline the reverse effect with a D_Leg_ significantly positive during spring of years 1 and 2 for the two-species mixtures. For spring in year 1, the positive D_Leg_, also measured for the five-species mixtures, appeared with very low *Trifolium* proportions (<5%). Despite we expected strong light competition induced by the grass species on *Trifolium* at the beginning of the experiment, the positive D_Leg_ underlines the existence of a strong facilitative effect of the grass on the legume species. This could be due to an indirect positive effect of the grass species associated, through lower soil N availability enabling N_2_ fixation initiation. Overall our data highlight the fact that the amount of positive interactions due to *Trifolium* presence could be partly driven by its proportion and its biomass amount in the mixture and thus by the plant–plant interaction outcomes.

## Conclusion

Our findings showed that observed complementarity effects leading to over-yielding were driven by traits related to water and N acquisition and use, as well as by *Trifolium* abundance in the community. Below-ground complementarity through root plasticity inducing a shift in resource uptake to deeper soil layers led to higher above-ground production, evapotranspiration and also higher WUE in mixtures containing *T. repens*. For temperate grasslands, albeit legumes proportion are known to fluctuate over time, our findings showed a strong positive effect of *Trifolium* on biomass production and diversity effect over a large range of legume abundance in mixture.

## Author Contributions

P-CC designed the study, HP and P-CC provided materials and method, performed analyses, and wrote the first draft of the manuscript.

## Conflict of Interest Statement

The authors declare that the research was conducted in the absence of any commercial or financial relationships that could be construed as a potential conflict of interest.

## References

[B1] Berendse F. (1979). Competition between plant populations with different rooting depths I. Theoretical considerations. *Oecologia* 43 19–26. 10.1007/BF00346669 28309824

[B2] Brooker R. W. Maestre F. T. Callaway R. M. Lortie C. L. Cavieres L. A. Kunstler G. (2008). Facilitation in plant communities: the past, the present, and the future. *J. Ecol.* 96 18–34. 10.1111/j.1365-2745.2007.01295.x

[B3] Caradus J. R. (1977). Structural variation of white clover root systems. *N. Z. J. Agric. Res.* 20 213–219. 10.1080/00288233.1977.10427325

[B4] Cardinale B. J. Wright J. P. Cadotte M. W. Carroll I. T. Hector A. Srivastava D. S. (2007). Impacts of plant diversity on biomass production increase through time because of species complementarity. *Proc. Natl. Acad. Sci.* 104 18123–18128. 10.1073/pnas.0709069104 17991772 PMC2084307

[B5] Craine J. M. Dybzinski R. (2013). Mechanisms of plant competition for nutrients, water and light. *Funct. Ecol.* 27 833–840. 10.1111/1365-2435.12081

[B6] De Boeck H. J. D. Lemmens C. M. H. M. Bossuyt H. Malchair S. Carnol M. Merckx R. (2006). How do climate warming and plant species richness affect water use in experimental grasslands? *Plant Soil* 288 249–261. 10.1007/s11104-006-9112-5

[B7] Díaz S. Lavorel S. de Bello F. Quétier F. Grigulis K. Robson T. M. (2007). Incorporating plant functional diversity effects in ecosystem service assessments. *Proc. Natl. Acad. Sci.* 104 20684–20689. 10.1073/pnas.0704716104 18093933 PMC2410063

[B8] Dimitrakopoulos P. G. Schmid B. (2004). Biodiversity effects increase linearly with biotope space. *Ecol. Lett.* 7 574–583. 10.1111/j.1461-0248.2004.00607.x

[B9] Dybzinski R. Fargione J. E. Zak D. R. Fornara D. Tilman D. (2008). Soil fertility increases with plant species diversity in a long-term biodiversity experiment. *Oecologia* 158 85–93. 10.1007/s00442-008-1123-x 18690478

[B10] Evans P. S. (1977). Comparative root morphology of some pasture grasses and clovers. *N. Z. J. Agric. Res.* 20 331–335. 10.1080/00288233.1977.10427343

[B11] Fargione J. Tilman D. (2005). Niche differences in phenology and rooting depth promote coexistence with a dominant C4 bunchgrass. *Oecologia* 143 598–606. 10.1007/s00442-005-0010-y 15791430

[B12] Feldman B. E. (2005). *Relative Importance and Value.* Rochester, NY: Social Science Research Network.

[B13] Frame J. (1986). The production and quality potential of four forage legumes sown alone and combined in various associations. *Crop Res.* 25 103–122.

[B14] Giller S. P. Hillebrand H. Berninger U.-G. O. Gessner M. Hawkins S. Inchausti P. (2004). Biodiversity effects on ecosystem functioning: emerging issues and their experimental test in aquatic environments. *Oikos* 104 423–436.

[B15] Grieu P. Lucero D. W. Ardiani R. Ehleringer J. R. (2001). The mean depth of soil water uptake by two temperate grassland species over time subjected to mild soil water deficit and competitive association. *Plant Soil* 230 197–209. 10.1023/A:1010363532118

[B16] Haynes R. J. (1980). Competitive aspects of the grass-legume association. *Adv. Agron.* 33 227–261. 10.1007/BF00225891

[B17] Hille Ris Lambers J. Harpole W. S. Tilman D. Knops J. Reich P. B. (2004). Mechanisms responsible for the positive diversity–productivity relationship in Minnesota grasslands. *Ecol. Lett.* 7 661–668. 10.1111/j.1461-0248.2004.00623.x

[B18] Hoekstra N. J. Finn J. A. Hofer D. Lüscher A. (2014). Do belowground vertical niche differences between deep- and shallow-rooted species enhance resource uptake and drought resistance in grassland mixtures? *Plant Soil* 394 21–34. 10.1007/s11104-014-2352-x

[B19] Høgh-Jensen H. Schjoerring J. K. (1997). Effects of drought and inorganic N form on nitrogen fixation and carbon isotope discrimination in Trifolium repens. *Plant Physiol. Biochem.* 35 55–62.

[B20] Jarvis S. C. Wilkins R. J. Pain B. F. (1996). Opportunities for reducing the environmental impact of dairy farming managements: a systems approach. *Grass Forage Sci.* 51 21–31. 10.1111/j.1365-2494.1996.tb02034.x

[B21] Kahmen A. Renker C. Unsicker S. B. Buchmann N. (2006). Niche complementarity for nitrogen: an explanation for the biodiversity and ecosystem functioning relationship? *Ecology* 87 1244–1255. 10.1890/0012-9658(2006)87[1244:NCFNAE]2.0.CO;2 16761603

[B22] Kutschera L. Lichtenegger E. (1992). *Wurzelatlas Mitteleuropäischer Grünlandpflanzen: Morphologie, Anatomie, Ökologie, Verbreitung, Soziologie, Wirtschaft.* Godfrey, IL: Gustav Fischer.

[B23] Leimer S. Kreutziger Y. Rosenkranz S. Beßler H. Engels C. Hildebrandt A. (2014). Plant diversity effects on the water balance of an experimental grassland. *Ecohydrology* 7 1378–1391. 10.1002/eco.1464

[B24] Leps J. De Bello F. Lavorel S. Berman S. (2006). Quantifying and interpreting functional diversity of natural communities: practical considerations matter. *Preslia* 78 481–501.

[B25] Loreau M. (1998). Separating sampling and other effects in biodiversity experiments. *Oikos* 82 600–602. 10.2307/3546381

[B26] Loreau M. Hector A. (2001). Partitioning selection and complementarity in biodiversity experiments. *Nature* 412 72–76. 10.1038/35083573 11452308

[B27] Loreau M. Naeem S. Inchausti P. Bengtsson J. Grime J. P. Hector A. (2001). Biodiversity and ecosystem functioning: current knowledge and future challenges. *Science* 294 804–808. 10.1126/science.1064088 11679658

[B28] Louault F. Pillar V. D. Aufrere J. Garnier E. Soussana J. F. (2005). Plant traits and functional types in responses to reduced disturbance in semi-natural grassland. *J. Veg. Sci.* 16 151–160. 10.1111/j.1654-1103.2005.tb02350.x

[B29] Marquard E. Weigelt A. Temperton V. M. Roscher C. Schumacher J. Buchmann N. (2009). Plant species richness and functional composition drive overyielding in a six-year grassland experiment. *Ecology* 90 3290–3302. 10.1890/09-0069.1 20120799

[B30] Mason N. W. H. Mouillot D. Lee W. G. Wilson J. B. (2005). Functional richness, functional evenness and functional divergence: the primary components of functional diversity. *Oikos* 111 112–118. 10.1111/j.0030-1299.2005.13886.x

[B31] Mokany K. Ash J. Roxburgh S. (2008). Functional identity is more important than diversity in influencing ecosystem processes in a temperate native grassland. *J. Ecol.* 96 884–893. 10.1111/j.1365-2745.2008.01395.x

[B32] Mommer L. Van Ruijven J. De Caluwe H. Smit-Tiekstra A. E. Wagemaker C. A. M. Joop Ouborg N. (2010). Unveiling below-ground species abundance in a biodiversity experiment: a test of vertical niche differentiation among grassland species. *J. Ecol.* 98 1117–1127. 10.1111/j.1365-2745.2010.01702.x

[B33] Mueller K. E. Tilman D. Fornara D. A. Hobbie S. E. (2013). Root depth distribution and the diversity–productivity relationship in a long-term grassland experiment. *Ecology* 94 787–793. 10.1890/12-1399.1

[B34] Nippert J. B. Holdo R. M. (2015). Challenging the maximum rooting depth paradigm in grasslands and savannas. *Funct. Ecol.* 29 739–745. 10.1111/1365-2435.12390

[B35] Nippert J. B. Knapp A. K. (2007). Soil water partitioning contributes to species coexistence in tallgrass prairie. *Oikos* 116 1017–1029. 10.1111/j.0030-1299.2007.15630.x

[B36] R Core Team (2009). *R: A Language and Environment for Statistical Computing.* Vienna: R Foundation for Statistical Computing. ISBN 3-900051-7-0.

[B37] Reich P. B. Tilman D. Isbell F. Mueller K. Hobbie S. Flynn D. (2012). Impacts of biodiversity loss escalate as redundancy fades. *Science* 336 589–592. 10.1126/science.1217909 22556253

[B38] Roscher C. Schumacher J. Gubsch M. Lipowsky A. Weigelt A. Buchmann N. (2012). Using plant functional traits to explain diversity–productivity relationships. *PLoS ONE* 7:e36760 10.1371/journal.pone.0036760 PMC3356333 22623961

[B39] Rosenkranz S. Wilcke W. Eisenhauer N. Oelmann Y. (2012). Net ammonification as influenced by plant diversity in experimental grasslands. *Soil Biol. Biochem.* 48 78–87. 10.1016/j.soilbio.2012.01.008

[B40] Schmid B. (2002). The species richness–productivity controversy. *Trends Ecol. Evol.* 17 113–114. 10.1016/S0169-5347(01)02422-2423

[B41] Schumacher J. Roscher C. (2009). Differential effects of functional traits on aboveground biomass in semi-natural grasslands. *Oikos* 118 1659–1668. 10.1111/j.1600-0706.2009.17711.x

[B42] Schwinning S. Weiner J. (1998). Mechanisms determining the degree of size asymmetry in competition among plants. *Oecologia* 113 447–455. 10.1007/s004420050397 28308024

[B43] Silvertown J. Dodd M. E. Gowing D. J. G. Mountford J. O. (1999). Hydrologically defined niches reveal a basis for species richness in plant communities. *Nature* 400 61–63.

[B44] Skinner R. H. Comas L. H. (2010). Root distribution of temperate forage species subjected to water and nitrogen stress. *Crop Sci.* 50 2178–2185. 10.2135/cropsci2009.08.0461

[B45] Skinner R. H. Sanderson M. A. Tracy B. F. Dell C. J. (2006). Above- and belowground productivity and soil carbon dynamics of pasture mixtures. *Agron. J.* 98:320 10.2134/agronj2005.0180a

[B46] Soussana J.-F. Tallec T. (2010). Can we understand and predict the regulation of biological N2 fixation in grassland ecosystems? *Nutr. Cycl. Agroecosyst.* 88 197–213. 10.1007/s10705-009-9335-y

[B47] Spehn E. M. Hector A. Joshi J. Scherer-Lorenzen M. Schmid B. Bazeley-White E. (2005). Ecosystem effects of biodiversity manipulations in european grasslands. *Ecol. Monogr.* 75 37–63. 10.1890/03-4101

[B48] Spehn E. M. Joshi J. Schmid B. Alphei J. Körner C. (2000). Plant diversity effects on soil heterotrophic activity in experimental grassland ecosystems. *Plant Soil* 224 217–230. 10.1023/A:1004891807664

[B49] Spehn E. M. Scherer-Lorenzen M. Schmid B. Hector A. Caldeira M. C. Dimitrakopoulos P. G. (2002). The role of legumes as a component of biodiversity in a cross-European study of grassland biomass nitrogen. *Oikos* 2 205–218. 10.1034/j.1600-0706.2002.980203.x

[B50] Stocker R. Körner C. Schmid B. Niklaus P. A. Leadley P. W. (1999). A field study of the effects of elevated CO2 and plant species diversity on ecosystem-level gas exchange in a planted calcareous grassland. *Glob. Chang. Biol.* 5 95–105. 10.1046/j.1365-2486.1998.00198.x

[B51] Suter M. Connolly J. Finn J. A. Loges R. Kirwan L. Sebastià M.-T. (2015). Nitrogen yield advantage from grass–legume mixtures is robust over a wide range of legume proportions and environmental conditions. *Glob. Chang. Biol.* 21 2424–2438. 10.1111/gcb.12880 25626994

[B52] Temperton V. M. Mwangi P. N. Scherer-Lorenzen M. Schmid B. Buchmann N. (2007). Positive interactions between nitrogen-fixing legumes and four different neighbouring species in a biodiversity experiment. *Oecologia* 151 190–205. 10.1007/s00442-006-0576-z 17048010

[B53] Tilman D. Knops D. Wedin D. Reich P. (2002). “Plant diversity and composition: effects on productivity and nutrient dynamics of experimental grasslands,” in *Biodiversity and Ecosystem Functioning. Synthesis and Perspective*, eds Loreau M. Naeem S. Inchausti P. (London: Oxford University Press), 21–35.

[B54] Vanelslander B. De Wever A. Van Oostende N. Kaewnuratchadasorn P. Vanormelingen P. Hendrickx F. (2009). Complementarity effects drive positive diversity effects on biomass production in experimental benthic diatom biofilms. *J. Ecol.* 97 1075–1082. 10.1111/j.1365-2745.2009.01535.x

[B55] Verheyen K. Bulteel H. Palmborg C. Olivié B. Nijs I. Raes D. (2008). Can complementarity in water use help to explain diversity–productivity relationships in experimental grassland plots? *Oecologia* 156 351–361. 10.1007/s00442-008-0998-x 18305961

[B56] von Felten S. Hector A. Buchmann N. Niklaus P. A. Schmid B. Scherer-Lorenzen M. (2009). Belowground nitrogen partitioning in experimental grassland plant communities of varying species richness. *Ecology* 90 1389–1399. 10.1890/08-0802.1 19537558

[B57] von Felten S. Schmid B. (2008). Complementarity among species in horizontal versus vertical rooting space. *J. Plant Ecol.* 1 33–41. 10.1093/jpe/rtm006

[B58] Zwicke M. Picon-Cochard C. Morvan-Bertrand A. Prud’homme M.-P. Volaire F. (2015). What functional strategies drive drought survival and recovery of perennial species from upland grassland? *Ann. Bot.* 116 1001–1015. 10.1093/aob/mcv037 25851134 PMC4640119

