# Supplementary material for: Presence of Trifolium repens Promotes Complementarity of Water Use and N Facilitation in Diverse Grass Mixtures
Source: Front Plant Sci. 2016 Apr 26;7:538. doi: 10.3389/fpls.2016.00538 (PMC4845251; doi:10.3389/fpls.2016.00538)
Supplement: Supplementary file 1 [file Data_Sheet_1.DOCX]

**Supplementary information**

**Presence of *Trifolium repens* promotes complementarity of water use and N facilitation in diverse grass mixtures**

HERNANDEZ Pauline, PICON-COCHARD Catherine

*(1) Supplementary Table and Figure*

*(2) Prediction of biomass production, net diversity and complementarity effects with CWM and functional diversity (FD_Q_) traits*

*(1) Supplementary Table and Figure*

Table S1: N content (%) measured in grass species present in the two and five-species mixtures either without (2-) or with (2+, 5+) the presence of *T. repens*. Mean values ± sem are shown by period and averaged over the whole experiment (Total, 113 y_1_-161 y_2_).

| Year | Periods | 2- | 2+ | 5+ | P-value |  |
| --- | --- | --- | --- | --- | --- | --- |
| 2013 | 113-143 | 3.94 ± 0.01 | 3.74 ± 0.03 | 4.21 ± 0.04 | 0.252 | |
| 2013 | 144-190 | 2.40 ± 0.02 | 2.71 ± 0.03 | 2.67 ± 0.11 | 0.238 | |
| 2013 | 191-224 | 2.49 ± 0.02 | 2.75 ± 0.04 | 2.59 ± 0.07 | 0.603 | |
| 2013 | 225-280 | 2.27 ± 0.02 | 2.55 ± 0.05 | 2.18 ± 0.05 | 0.714 | |
| 2014 | 281-101 | 2.45 ± 0.02 | 3.14 ± 0.07 | 2.53 ± 0.06 | 0.298 | |
| 2014 | 102-161 | 1.62 ± 0.01 b | 2.48 ± 0.05 a | 1.69 ± 0.07 ab | **0.049** | |
| Total | 113 y_1_-161 y_2_ | 2.53 ± 0.06 | 2.91 ± 0.13 | 2.65 ± 0.04 | 0.362 | |

For each period, if present different letters correspond to statistical differences (P ≤ 0.05). NS: P > 0.05.

Figure S1: Temporal dynamics of soil water content measured at 15, 30 and 50 cm, for monocultures without (1-) or with legume (1+), two-species mixtures without (2-) and with (2+) legume and five-species mixture with legume (5+). Daily averages are shown. For 1+ sward, no data are shown from DOY 166 because of probe failure.

*(2) Prediction of biomass production, net diversity and complementarity effects with CWM and functional diversity (FD_Q_) traits*

Trait composition effects on ecosystem functioning can be assessed by calculating two main indices (a) trait community weighted mean, quantifying the dominant trait values in a community (Garnier et al., 2004) and (b) functional diversity. The Functional Diversity (FD) is considered as the extent of functional trait variation among the species in a community (Petchey and Gaston, 2002). The FD can also be defined as the overall difference among species in a community in terms of their traits. Trait community weighted mean (CWM) is closely related to the mass ratio hypothesis (Grime, 1998) which suggested that the functional traits of dominant species in a community (functional identity) are the main determinant of ecosystem processes. CWM can therefore be related to selection effect. Furthermore, the FD recently appeared as an important issue because it is based on the assumption that the diversity in resource use strategies increases with increasing trait dissimilarity among species. The FD is therefore closely related to complementarity effect (Mason et al., 2005).

The two components of net biodiversity effect, selection and complementarity effects, can be obtained using the additive partitioning method (Loreau and Hector, 2001). Thus, complementarity effect, including both facilitation and species-complementary resource use, has been proposed as main mechanism explaining over-yielding (Cardinale et al., 2007). While the concept of species-complementary resource use can easily be related to FD of traits, it remains unclear how the facilitation concept can be more related to the CWM rather than the FD of traits, or conversely. Recent studies looked for assess the relative importance of the two components of trait community, the traits identity (CWM) and diversity (FD) of the community, as drivers of ecosystem functioning. Indeed, the CWM and FD of trait values have been shown to jointly explain variation in aboveground productivity in semi-natural grasslands (Díaz et al., 2007; Mokany et al., 2008; Schumacher and Roscher, 2009). In experimental grasslands, studies on the relative importance of the CWM and FD of traits values as drivers of above-ground production showed contrasted results, with a higher community biomass correlated positively with functional trait diversity (Cadotte et al., 2009; Wacker et al., 2009) or with a combination of functional trait diversity and community-weighted means of trait values (Mouillot et al., 2011; Roscher et al., 2012). Also, using CWM and FD indices as predictors for community biomass production, net diversity and complementarity effects can lead to the identification of relevant traits, which is essential for a mechanistic understanding of the role of plant diversity.

To study the relative contribution of CWM and FD of trait values to the amount of explained variation in above-ground biomass production and diversity effects, we considered statistical models with different combinations of explanatory variables: (a) CWM, (b) FD, (c) CWM and FD (Roscher et al., 2012). We performed these analyses on the two- and five-species mixtures using six variables measured at species level. Root trait (R.growth) and traits related to water use (Nyield/ET, REW, WUE), only measured at the community level, could not be included. Thus, functional trait diversity was computed as Raòs quadratic entropy (FD_Q,_ Leps et al., 2006) on Biom st1, δ^13^C, H.growth, L.area, LDMC and N. The following equation was used: $\mathrm{FD}_{Q} = \sum_{i=1}^{S} \sum_{j=1}^{S} p_{i}p_{j}d_{ij}$, where *S* is the number of species in the community, *p_i_* and *p_j_* are the relative abundances of species *i* and *j*, and *d_ij_* is the trait distance between species *i* and *j* in the community; *p_i_* and *p_j_* are defined as (i) the species proportions in total biomass for LDMC, L.area, Biom st1 and H.growth, (ii) the species green leaf proportions for N, (iii) the species leaf area proportions for δ^13^C. The distance *d_ij_*, also called species dissimilarity, is scaled between zero (no dissimilarity) and one (maximum dissimilarity). Within each class of models, we selected, the best fit based on leave-one-out cross validation (R packages car and leaps). The coefficient of determination R^2^ is given as a summary measure for explained variation. The final selected models contain five traits, as adding additional variables did not significantly increase R². We also studied the relative importance of each variable within each model selected using the proportional marginal variance decomposition metric proposed by Feldman (2005), which can be interpreted as a weighted average over orderings among regressors, with data-dependent weights (R package relaimpo). Moreover, standardized PCAs were applied to explore multiple relationships between the CWM and / or FD_Q_ traits. For PCAs and model selection, average data for the whole experiment and summer of Year 2 (period 102-161) are shown, owing to a more pronounced effect of legume presence at the end of the experiment.

Considering the scale of the whole experimental period (20 months), in separate models based on six predictors, CWM explained a larger proportion of variation in community biomass (*R*² = 0.822), net effect (NE, *R*² = 0.714) and complementarity effect (CE, *R*² = 0.723) than models based on FD_Q_ (respectively *R*² = 0.688, 0.673 and 0.727) (Table S2a). Moreover, the proportion of explained variation increased in models that included both CWM and FD_Q_ (*R*² = 0.906 for biomass, *R*² = 0.831 for NE and *R*² = 0.870 for CE; Table S2a) compared with models based purely on CWM or FD_Q_. Partial *R*² values also provided evidence that CWM was the most important group of predictor variables in the combined model. A similar and even more pronounced pattern occurred for the diversity effects and community biomass produced during summer in Year 2. Indeed, in separate models based on six predictors, CWM explained a larger proportion of variation in community biomass (*R*² = 0.949), net effect (NE, *R*² = 0.859) and also complementarity effect (CE, *R*² = 0.757) than models based on FD_Q_ (respectively *R*² = 0.572, 0.590 and 0.583). Then partial *R*² values in models that included both CWM and FD_Q_ showed an even greater dominance of CWM traits over FD_Q_ traits (Table S2b)_._ This is consistent with the results of PCAs on the average data for the whole experiment, which show a better discrimination of mixture types (especially grass-*Trifolium vs*. grass mixtures) along the different axes in analyses incorporating CWM and FD_Q_ than in those based on a single group of predictors (Figure S2). Moreover, in separate PCAs, mixtures types were better discriminated with CWM of traits rather than FD_Q_ (Figure S2). Thus, the finding of models selection, combined with PCAs, suggest that functional identity had the largest effects on community biomass production and diversity effects.

Our analyses of CWM and FD_Q_ based on 6 functional traits confirm the results of Roscher et al. (2012), who show that in all cases the combination of CWM and FD_Q_ results in models with the greatest explanatory power for variation in biomass production and measurements of diversity effects. Also, CWM component explained a greater proportion of variation in community biomass and net diversity effect than FD_Q_, whereas in theory the CWM of traits values should be more related with the selection effect rather than others biodiversity components. Moreover, our results show that CWM of traits explained a greater proportion of complementarity effect than FD_Q_, despite the increased contribution of functional diversity (29 %) compared with those for biomass or net effect. Despite the concerns on the limited number of variables incorporated in the model selection for the CWM and FD_Q_ comparison, our findings that show a best explanatory power of CWM traits can be validate to a certain extent according to their consistence with Roscher et al. (2012). Thus, given the observed importance of CWM predictors, we focused our model selection analyses on 10 CWM of traits values linked to light, N and water resources. Indeed, we secondly tried to identify which leaf and root traits better explain biomass, over-yielding and complementarity effect measured in mixture, and especially to determine the relative importance of resources involved in these responses.

Table S2a: Summary of best statistical models based on different groups of predictor variables (community-weighted means of trait values (CWM) and functional trait diversity (FD_Q_)) for the whole experiment community biomass, net biodiversity effects and complementarity effects.

| Whole experiment | | *R*^2^ | | 6 traits: CWM | |  | | | | |  | | | |  | | | |  | | | | |  | | |  |  |  |  |  |  |
| --- | --- | --- | --- | --- | --- | --- | --- | --- | --- | --- | --- | --- | --- | --- | --- | --- | --- | --- | --- | --- | --- | --- | --- | --- | --- | --- | --- | --- | --- | --- | --- | --- |
| Biomass | **0.822** | | Selected traits | | | | L.area | | | Biom st1 | | | | N | | | | LDMC | | | | δ^13^C | | | | |  |  |  |  |  |  |
|  |  | | % *R*^2^ | | | | 81.4 | | | 17.0 | | | | 1.0 | | | | 0.5 | | | | 0.1 | | | | |  |  |  |  |  |  |
| Net biodiversity | **0.714** | | Selected traits | | | | L.area | | | δ^13^C | | | | LDMC | | | | H.growth | | | | N | | | | |  |  |  |  |  |  |
| effect |  | | % *R*^2^ | | | | 93.8 | | | 2.8 | | | | 2.8 | | | | 0.3 | | | | 0.3 | | | | |  |  |  |  |  |  |
| Complementarity | **0.723** | | Selected traits | | | | L.area | | | LDMC | | | | H.growth | | | | δ^13^C | | | | N | | | | |  |  |  |  |  |  |
| effect |  | | % *R*^2^ | | | | 95.4 | | | 2.2 | | | | 1.2 | | | | 1.0 | | | | 0.2 | | | | |  |  |  |  |  |  |
| Whole experiment | R^2^ | | 6 traits: FD_Q_ | | | |  | | |  | | | |  | | | |  | | | |  | | | | |  |  |  |  |  |  |
| Biomass | **0.688** | | Selected traits | | | | Biom st1 | | | N | | | | δ^13^C | | | | L.area | | | | | LDMC | | | |  |  |  |  |  |  |
|  |  | | % *R*^2^ | | | | 70.6 | | | 23.0 | | | | 4.0 | | | | 2.1 | | | | | 0.2 | | | |  |  |  |  |  |  |
| Net biodiversity | **0.673** | | Selected traits | | | | Biom st1 | | | H.growth | | | | LDMC | | | | L.area | | | | | δ^13^C | | | |  |  |  |  |  |  |
| effect |  | | % *R*^2^ | | | | 77.3 | | | 7.9 | | | | 7.4 | | | | 7.0 | | | | | 0.5 | | | |  |  |  |  |  |  |
| Complementarity | **0.727** | | Selected traits | | | | Biom st1 | | | H.growth | | | | L.area | | | | LDMC | | | | | δ^13^C | | | |  |  |  |  |  |  |
| effect |  | | % *R*^2^ | | | | 78.1 | | | 11.1 | | | | 4.8 | | | | 4.6 | | | | | 1.5 | | | |  |  |  |  |  |  |
| Whole experiment | R^2^ | | 6 traits: CWM | |  | | |  |  | | | | Partial  *R*^2^ | | | | 6 traits: FD_Q_ | | | |  | | | | |  | | |  | | Partial  *R*^2^ | |
| Biomass | **0.906** | | Selected traits | | L.area | | | Biom st1 | H.growth | | | **0.787** | | | | Selected traits | | | | L.area | | | | | H.growth | | |  | | **0.120** | |  |
|  |  | | % *R*^2^ | | 75.3 | | | 10.8 | 0.7 | | |  | | | | % *R*^2^ | | | | 9.3 | | | | | 3.8 | | |  | |  | |  |
| Net biodiversity | **0.831** | | Selected traits | | L.area | | | N | δ^13^C | | | **0.695** | | | | Selected traits | | | | H.growth | | | | | L.area | | |  | | **0.136** | |  |
| effect |  | | % *R*^2^ | | 75.5 | | | 5.8 | 2.3 | | |  | | | | % *R*^2^ | | | | 8.5 | | | | | 8.0 | | |  | |  | |  |
| Complementarity | **0.870** | | Selected traits | | L.area | | | N |  | | | **0.580** | | | | Selected traits | | | | Biom st1 | | | | | L.area | | | δ^13^C | | **0.291** | |  |
| effect |  | | % R^2^ | | 63.8 | | | 2.8 |  | | |  | | | | % R^2^ | | | | 23.8 | | | | | 6.5 | | | 3.2 | |  | |  |

Table S2b. Summary of best statistical models based on different groups of predictor variables (community-weighted means of trait values (CWM) and functional trait diversity (FD_Q_)) for summer Year 2 community biomass, net biodiversity effects and complementarity effects.

| Summer Year 2 | R^2^ | 6 traits: CWM | |  | | |  | | | |  |  |  |  |  |  |  |
| --- | --- | --- | --- | --- | --- | --- | --- | --- | --- | --- | --- | --- | --- | --- | --- | --- | --- |
| Biomass | **0.949** | Selected traits | | | L.area | | Biom st1t | | | LDMC | | H.growth | δ^13^C |  |  |  |  |
|  |  | % *R*^2^ | | | 97.7 | | 1.7 | | | 0.4 | | 0.2 | 0.1 |  |  |  |  |
| Net biodiversity | **0.859** | Selected traits | | | L.area | | N | | | Biom st1 | | δ^13^C | H.growth |  |  |  |  |
| effect |  | % *R*^2^ | | | 92.3 | | 5.4 | | | 1.1 | | 1.0 | 0.1 |  |  |  |  |
| Complementarity | **0.757** | Selected traits | | | L.area | | Biom st1 | | | N | | δ^13^C | H.growth |  |  |  |  |
| effect |  | % *R*^2^ | | | 91.7 | | 4.0 | | | 3.0 | | 0.9 | 0.5 |  |  |  |  |
| Summer Year 2 | R^2^ | 6 traits: FD_Q_ | | |  | |  | | |  | |  |  |  |  |  |  |
| Biomass | **0.572** | Selected traits | | | L.area | | δ^13^C | | | Biom st1 | | LDMC | N |  |  |  |  |
|  |  | % *R*^2^ | | | 80.8 | | 12.2 | | | 5.2 | | 1.7 | 0.1 |  |  |  |  |
| Net biodiversity | **0.590** | Selected traits | | | L.area | | Biom st1 | | | δ^13^C | | LDMC | N |  |  |  |  |
| effect |  | % *R*^2^ | | | 77.5 | | 10.7 | | | 8.1 | | 2.6 | 1.1 |  |  |  |  |
| Complementarity | **0.583** | Selected traits | | | L.area | | Biom st1 | | | δ^13^C | | H.growth | LDMC |  |  |  |  |
| effect |  | % *R*^2^ | | | 66.3 | | 17.7 | | | 8.6 | | 8.7 | 1.5 |  |  |  |  |
| Summer Year 2 | R^2^ | 6 traits: CWM |  | | |  |  |  | | |  | Partial R^2^ | 6 traits: FD_Q_ |  |  |  | Partial *R*^2^ |
| Biomass | **0.977** | Selected traits | L.area | | | LDMC | H.growth | |  | | | **0.943** | Selected traits | Biom st1 | δ^13^C |  | **0.034** |
|  |  | % *R*^2^ | 94.2 | | | 1.4 | 0.9 | |  | | |  | % *R*^2^ | 3.1 | 0.5 |  |  |
| Net biodiversity | **0.932** | Selected traits | L.area | | | N | H.growth | | LDMC | | | **0.841** | Selected traits | Biom st1 |  |  | **0.090** |
| effect |  | % *R*^2^ | 83.4 | | | 6.5 | 0.3 | | 0.1 | | |  | % *R*^2^ | 9.6 |  |  |  |
| Complementarity | **0.878** | Selected traits | L.area | | | LDMC | Biom st1 | |  | | | **0.767** | Selected traits | H.growth | Biom st1 |  | **0.112** |
| Effect |  | % *R*^2^ | 77.3 | | | 0.6 | 0.4 | |  | | |  | % *R*^2^ | 17.8 | 3.9 |  |  |

Figure S2: Standardized principal component analyses (PCAs) combining 6 functional traits for mixtures of two and five species: community-weighted means of trait values (CWM, left), functional trait diversity (FD, center) and both CWM and FD (right), averaged over the experiment period. Abbreviations are as follows: Biom st1, percentage of biomass in the top canopy layer (%), δ^13^C: leaf C isotopic composition (‰), H.growth: growth height (cm day^-1^), L.area: leaf area (m^2^ pot^-1^), LDMC: leaf dry matter content (mg g^-1^), N: N community weighted mean (%).


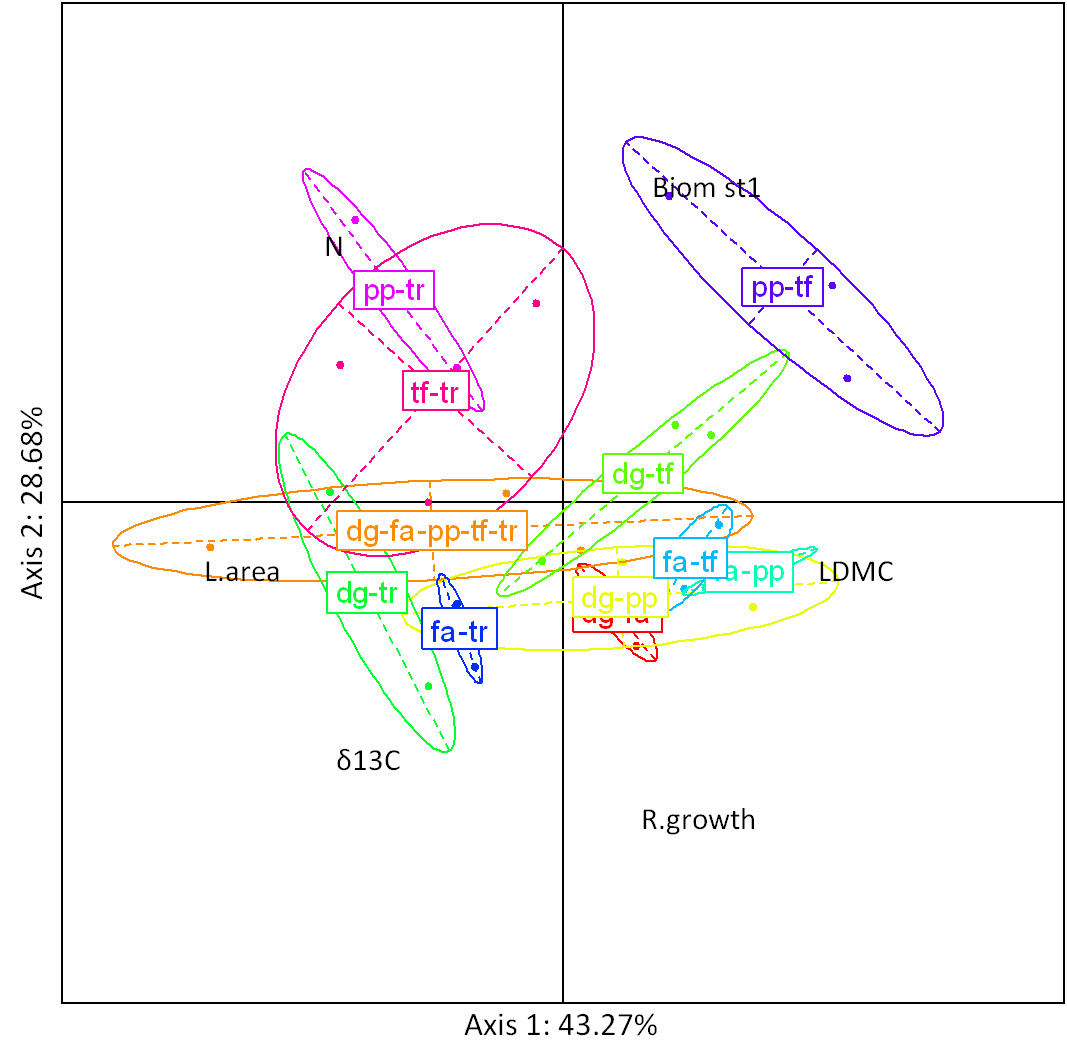

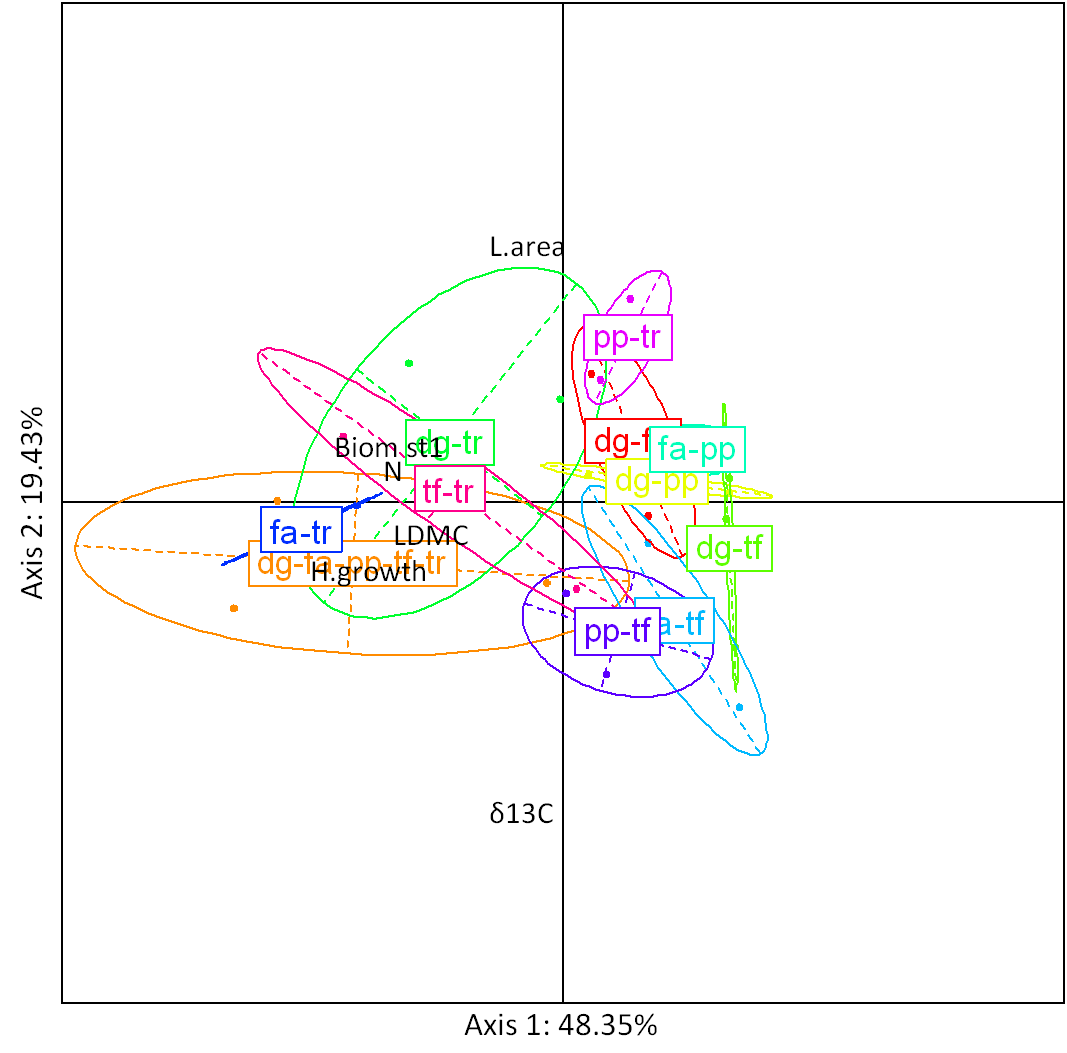

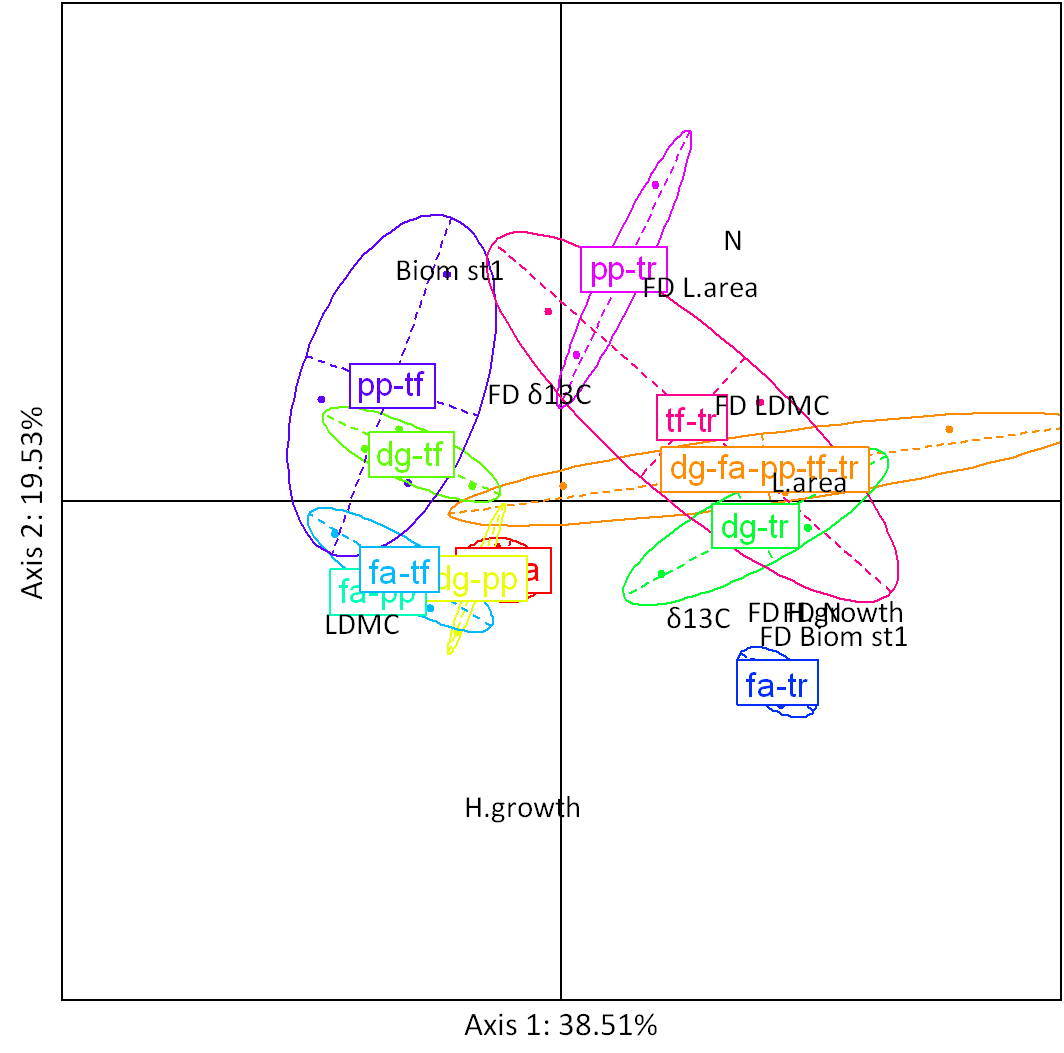


Cadotte, M. W., Cavender-Bares, J., Tilman, D., and Oakley, T. H. (2009). Using Phylogenetic, Functional and Trait Diversity to Understand Patterns of Plant Community Productivity. *PLoS ONE* 4, e5695. doi:10.1371/journal.pone.0005695.

Cardinale, B. J., Wright, J. P., Cadotte, M. W., Carroll, I. T., Hector, A., Srivastava, D. S., et al. (2007). Impacts of plant diversity on biomass production increase through time because of species complementarity. *Proc. Natl. Acad. Sci.* 104, 18123–18128. doi:10.1073/pnas.0709069104.

Díaz, S., Lavorel, S., de Bello, F., Quétier, F., Grigulis, K., and Robson, T. M. (2007). Incorporating plant functional diversity effects in ecosystem service assessments. *Proc. Natl. Acad. Sci. U. S. A.* 104, 20684–20689. doi:10.1073/pnas.0704716104.

Feldman, B. E. (2005). Relative Importance and Value. Rochester, NY: Social Science Research Network Available at: http://papers.ssrn.com/abstract=2255827 [Accessed October 20, 2015].

Garnier, E., Cortez, J., Billès, G., Navas, M.-L., Roumet, C., Debussche, M., et al. (2004). Plant functional markers capture ecosystem properties during secondary succession. *Ecology* 85, 2630–2637. doi:10.1890/03-0799.

Grime, J. P. (1998). Benefits of plant diversity to ecosystems: immediate, filter and founder effects. *J. Ecol.* 86, 902–910. doi:10.1046/j.1365-2745.1998.00306.x.

Leps, J., De Bello, F., Lavorel, S., and Berman, S. (2006). Quantifying and interpreting functional diversity of natural communities: practical considerations matter. *Preslia* 78, 481–501.

Loreau, M., and Hector, A. (2001). Partitioning selection and complementarity in biodiversity experiments. *Nature* 412, 72–76. doi:10.1038/35083573.

Mason, N. W. H., Mouillot, D., Lee, W. G., and Wilson, J. B. (2005). Functional richness, functional evenness and functional divergence: the primary components of functional diversity. *Oikos* 111, 112–118. doi:10.1111/j.0030-1299.2005.13886.x.

Mokany, K., Ash, J., and Roxburgh, S. (2008). Functional identity is more important than diversity in influencing ecosystem processes in a temperate native grassland. *J. Ecol.* 96, 884–893. doi:10.1111/j.1365-2745.2008.01395.x.

Mouillot, D., Villéger, S., Scherer-Lorenzen, M., and Mason, N. W. H. (2011). Functional Structure of Biological Communities Predicts Ecosystem Multifunctionality. *PLoS ONE* 6, e17476. doi:10.1371/journal.pone.0017476.

Petchey, O. L., and Gaston, K. J. (2002). Functional diversity (FD), species richness and community composition. *Ecol. Lett.* 5, 402–411. doi:10.1046/j.1461-0248.2002.00339.x.

Roscher, C., Schumacher, J., Gubsch, M., Lipowsky, A., Weigelt, A., Buchmann, N., et al. (2012). Using Plant Functional Traits to Explain Diversity–Productivity Relationships. *PLoS ONE* 7, e36760. doi:10.1371/journal.pone.0036760.

Schumacher, J., and Roscher, C. (2009). Differential effects of functional traits on aboveground biomass in semi-natural grasslands. *Oikos* 118, 1659–1668. doi:10.1111/j.1600-0706.2009.17711.x.

Wacker, L., Baudois, O., Eichenberger-Glinz, S., and Schmid, B. (2009). Diversity effects in early- and mid-successional species pools along a nitrogen gradient. *Ecology* 90, 637–648.
